# Genotoxic and Hematological Effects Associated with Chronic Dietary Mercury Toxicity in Juvenile Tilapia *Oreochromis* sp.

**DOI:** 10.1021/acsomega.4c09749

**Published:** 2025-02-20

**Authors:** Ahieska A. Liscano-Carreño, Cassiano J. Saatkamp, Ricardo B. de Oliveira, Luís R. R. Rodrigues

**Affiliations:** † Programa de Pós-Graduação em Biodiversidade e Biotecnologia (REDE BIONORTE), Instituto de Saúde Coletiva (ISCO), Universidade Federal do Oeste do Pará (UFOPA), Rua Vera Paz, s/no., Salé, CEP, 68040-255 Santarém, Pará, Brazil; ‡ Laboratório de Genética & Biodiversidade (LGBio), Instituto de Ciências da Educação (ICED), Universidade Federal do Oeste do Pará (UFOPA), Rua Vera Paz, s/no, Salé, CEP, 68040-255 Santarém, Pará, Brazil; § Departamento de Biología, Universidad de Oriente (UDO), Avenida Universidad, s/no, Cod Postal 6101, Cumaná, Sucre, Venezuela; ∥ Laboratório Santos, 68040-255 Santarém, Pará, Brasil; ⊥ Instituto de Ciências da Educação (ICED), Universidade Federal do Oeste do Pará (UFOPA), Rua Vera Paz, s/no, Salé, CEP, 68040-255 Santarém, Pará, Brazil

## Abstract

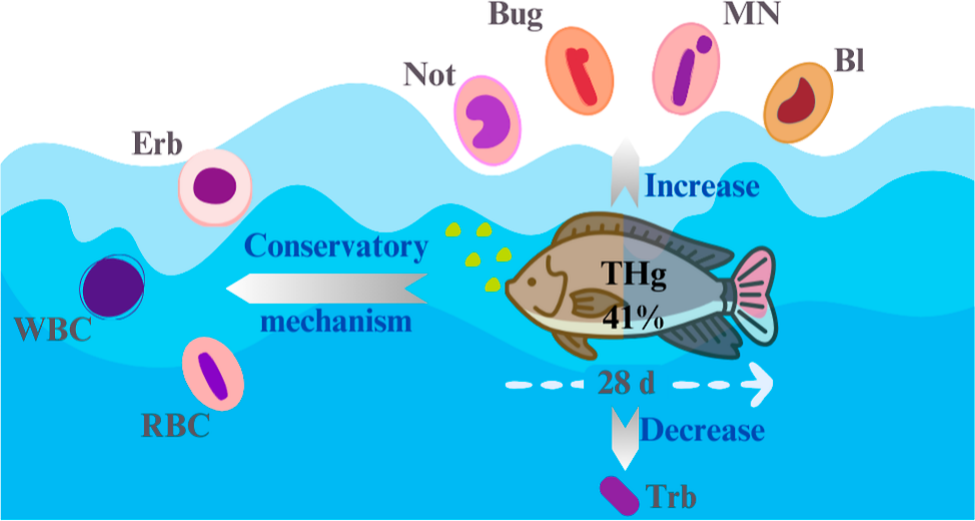

Fish farming and artisanal fishing represent important protein sources for riverside communities and populations of small towns in the Amazon. In recent decades, the Amazon basin has been the target of environmental contamination by mercury (Hg), which warns of possible adverse effects of human exposure through food. In this study, we evaluated the effect of mercury bioaccumulation in juvenile tilapia exposed via dietary intake. The fish were fed commercial feed supplemented with methylmercury chloride for a period of 28 days. Hematological parameters (hemogram, hematocrit (Hct), hemoglobin (Hgb), mean corpuscular volume (MCV), mean corpuscular hemoglobin (MCH), mean corpuscular hemoglobin concentration (MCHC)) and genotoxic effects in blood (micronucleus (MN), erythrocytic nuclear abnormalities (ENAs), DNA damage) were analyzed. Total mercury (THg) was determined in muscle tissue and blood. Hg bioaccumulation increased 7-fold in exposed fish, representing a body accumulation rate of 41%. No variation in growth performance or feeding habits was observed. The following biomarkers Hgb, thrombocytes (Trb), MCH, MCHC, MN and ENAs showed variation as a function of exposure time. Compensatory mechanisms of defense metabolism showed greater deficiency between 21 and 28 days.

## 1 Introduction

Aquaculture is one of the fastest growing food industries in the world, accounting for around 50% of the fish consumed globally; supporting fishery production since 1970, with the introduction of *Oreochromis* sp. from Africa to several countries with the aim of promoting food security, improving genetic lines over time and thus their performance in high densities, reduced spaces and even without affecting the behavior of the fish, achieving its full zootechnical and economic potential.^1−4^ Tilapia farming is one of the main culture, consumption and import items in Brazil, produced in intensive systems, achieving in 2022 an increase of 29.05% in production compared to the previous year, with Oreochromis sp being the most produced species (99.38% of the total volume); however, in the North region, there has been a boom in the implementation of breeding of this species on fish farms;^4−7^ contributing significantly to the supply of proteins in riverside communities and small towns in the Amazon region.

The expansion of tilapia culture in the Amazon, specifically in the Tapajós River basin, which is known for its long history of water and sediment contamination with Hg released from gold mining, forest deforestation and burning activities,^8−11^ points to the need for environmental monitoring and health surveillance, as human exposure to mercurial contamination through ingestion of contaminated fish has been treated as a serious problem documented in recent decades in this region. Mercury, once disposed of into soils or discharged in the aquatic environment by mining activity, as well as its availability through soil erosion resulting from deforestation, becomes available to the biota in general through methylation processes carried out by microorganisms, triggering bioaccumulation and biomagnification processes in the food chain, which leads to the accumulation of the metal in fish tissues, posing risks to both human health and ecosystems.^11−14^

Many species of fish in the Amazon (mainly in piscivores, although specific cases have been reported in species belonging to lower trophic levels) presented mercury levels above the safety limit established by the World Health Organization (WHO) showing high hazard coefficients, recommending a decrease in the daily consumption rate for these species.^12,14−17^ Chronic exposure to mercury has been linked to a number of health problems, including neurological, kidney, intestinal damage, among others. In addition, genotoxicological studies indicate that prolonged exposure to mercury can result in DNA damage, increasing the risk of mutations.^12,16,18^

Hematological biomarkers are widely used as indicators of the general health of aquatic organisms, allowing the assessment of environmental and toxicological impacts. Hematological changes, such as variations in erythrocyte and leukocyte counts in addition to cellular and nuclear abnormalities in erythrocytes (genotoxicity), are frequently observed in fish exposed to contaminants and heavy metals, among them the Hg, due to membrane damage and the high permeability of these toxins as the main causes of these effects.^19−25^ The most widely used biomarkers of genotoxicity in toxicity studies, as well as in environmental monitoring, are the micronucleus test and erythrocyte nuclear anomalies (ENAs), which, as the term MN test, has evolved into the micronucleus cytome assay; besides, nuclear anomalies they can be classified according to the degree and characteristic of the deformation of the nuclear membrane or arrangement of the chromatin within it, commonly identifying the forms: binucleated (Bn), blebbed (Bl), bud (Bud), notched (Not), lobbed (Lob); we also find single-cell electrophoresis known as the comet assay (CA) evaluated mainly in fish erythrocytes but not exclusively.^26−31^ In the present study we sought to determine the possible effects on the blood count and the genotoxic effects on the blood of fish exposed to a diet enriched with mercury. Monitoring of fish health should be a good practice in order to attain food security and resilience for traditional riverine communities, in congruence with the Global Sustainable Development Goals.^32^

## 2 Results

### 2.1 Growth Performance Analysis

A slight weight gain was observed in both experimental groups, without distinguishing differences or negative effects due to mercury (0.5 mg kg^–1^) as well as in the different growth performance variables [weight gain (WG, g), daily weight gain (DWG, g day^–1^), weight gain percent (WG %), specific growth rate (SGR) and feed conversion ratio (FCR)] at 28 days of exposure did not present differences between groups (ANOVA: 0.18; 0.07; 0.03; 0.06; 0.76 *p* > 0.05, respectively) (Table 1).

**Table 1:** Effects of Methylmercury (0.5 mg kg^–1^) in the Diet for 28 days of Exposure on Growth Performance Variables of Juvenile Tilapia

	W (*t*_0_)	W (*t*_28_)	GW (g)	DWG (g dia^–1^)	WG %	SGR	FCR
CG	15.66 ± 5.45	19.59 ± 5.85	2.80 ± 1.44	20.40 ± 5.65	17.77 ± 12.41	0.58 ± 0.35	6.41 ± 3.10
EG	15.47 ± 4.66	18.96 ± 5.76	3.48 ± 1.37	18.11 ± 5.60	22.53 ± 8.20	0.72 ± 0.23	5.07 ± 1.88

### 2.2 Determination of Total Mercury and Accumulation Rate

**Figure 1:**
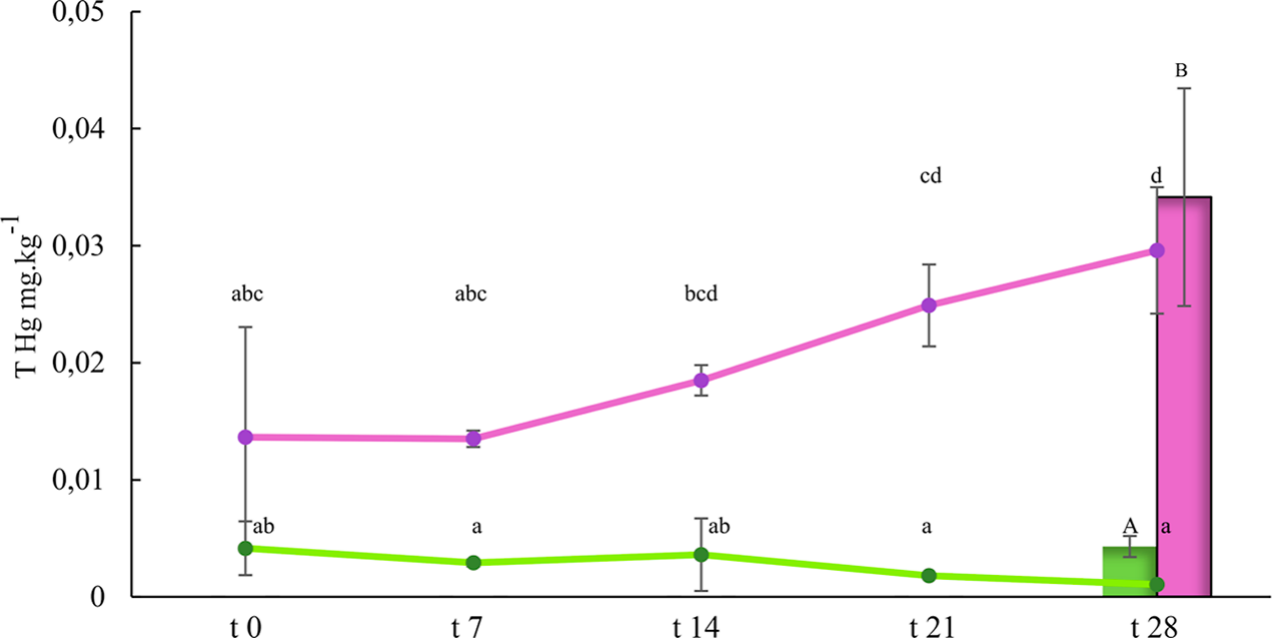
Mercury concentration mg kg^–1^ in juvenile tilapia fed with a diet enriched with methylmercury (0.5 mg kg^–1^). THg concentration in blood at 0, 7, 14, 21, and 28 days (line graph), THg concentration in muscle at day 28 (bar graph). Lowercase and uppercase superscript letters indicate group differences in blood THg and muscle THg, respectively (*p* < 0.001). CG (■(green)), EG (■(purple)).

The mean blood mercury concentration of the control group did not vary significantly during the experiment (*t*_0_ = 0.004 ± 0.002 mg kg^–1^; *t*_28_ = 0.001 ± 0.0002 mg kg^–1^). However, an increase in the blood mercury concentration of the exposed group was observed (*t*_0_ = 0.01 ± 0.009 mg kg^–1^; *t*_28_ = 0.03 ± 0.005 mg kg^–1^). The variation in blood mercury concentration was significant for both experimental groups at the end of the experiment (ANOVA: 14.47; *p* < 0.001). Likewise, mercury bioaccumulation in muscle tissue also varied significantly between the two groups (KW = 11.36 *p* < 0.001), with an increase of up to 7-fold in the exposed group, which represents a body accumulation rate of 41% (Figure 1).

### 2.3 Hematological Biomarkers

**Figure 2:**
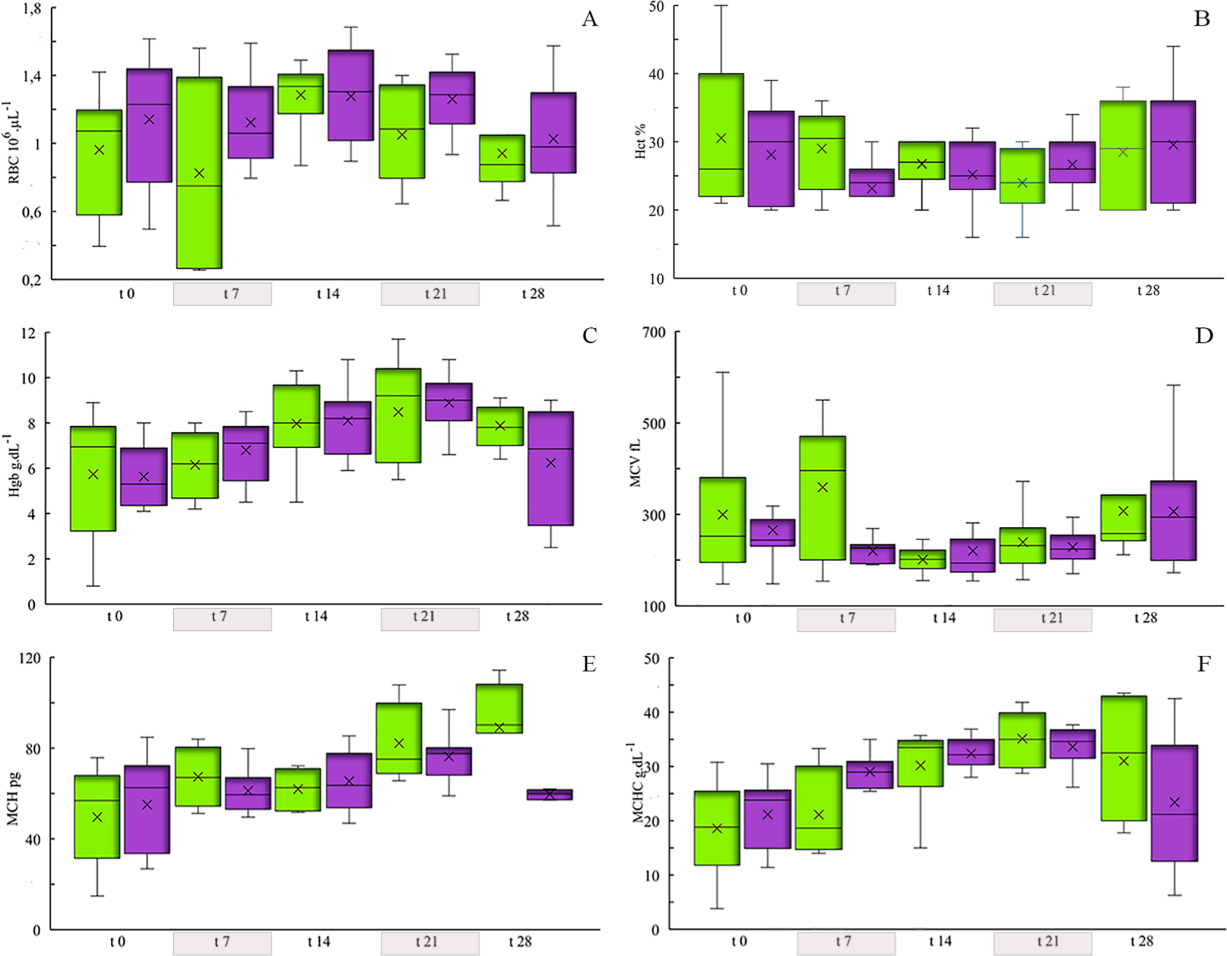
Erythrogram with hematimetric indices in juvenile tilapia with a diet enriched with methylmercury (0.5 mg kg^–1^). In the graph, the rectangular box shows the range of the data divided by a segment that indicates the median, the x represents the mean, and the vertical bar shows the dispersion of the data with the minimum and maximum values. Exposure time at 0, 7, 14, 21, and 28 days. CG (■(green)), EG (■(purple)). (A) RBC (cell 10^6^ μL^–1^), (B) Hct (%), (C) Hgb (g dL^–1^), (D) MCV (fL), (E) MCH (pg), (F) MCHC (g dL^–1^).

**Figure 3:**
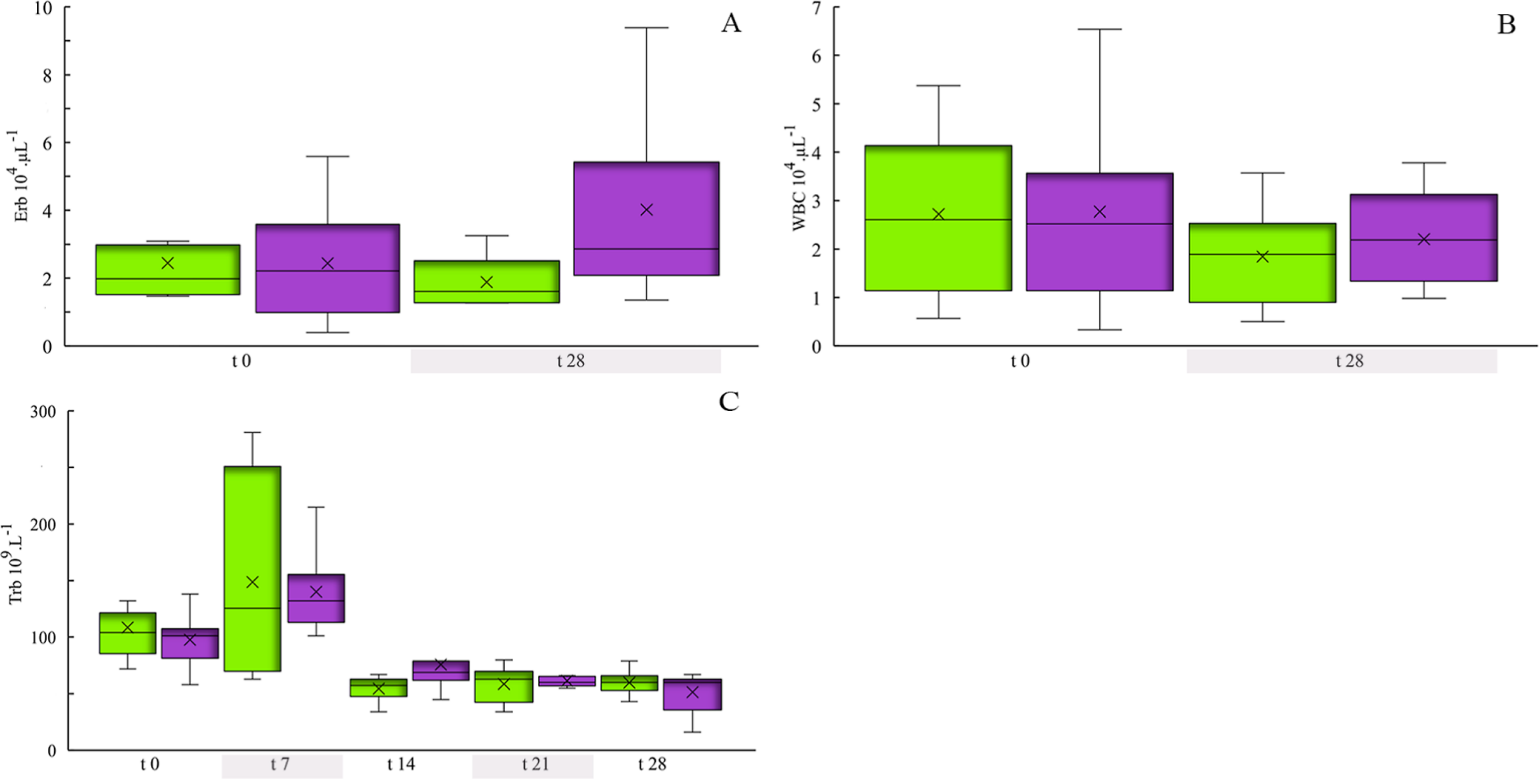
Leukocyte, erythroblast and thrombocyte counts of tilapia over time, exposed to methylmercury (0.5 mg kg^–1^). In the graph, the rectangular box shows the range of the data divided by a segment that indicates the median, the x represents the mean, and the vertical bar shows the dispersion of the data with the minimum and maximum values. Exposure time at 0, 7, 14, 21, and 28 days. CG (■(green)), EG (■(purple)). (A) Erb (cell 10^4^ μL^–1^), (B) WBC (cell 10^4^ μL^–1^), (C) Trb (cell 10^9^ L^–1^).

There was no variation in erythrocyte count (RBC), hematocrit (Hct) and mean corpuscular volume (MCV), however, Hemoglobin (Hgb) had mean values with significant variation between groups and throughout the exposure time (ANOVA: 3.45 *p* < 0.01) (Figure 2a,b,d,c, respectively). The highest Hgb values were recorded at *t*_21_ (CG: 8.49 ± 2.27 g dL^–1^; EG: 8.89 ± 1.24 g dL^–1^). Similarly, the MCH index showed a significant variation between groups throughout the experiment (ANOVA: 4.68 *p* < 0.001), with the control group registering an increase until the end (*t*_28_), while the exposed group registered an increase until *t*_21_ (MCH = 76.26 ± 11.12 pg) with a reduction in the last sampling at *t*_28_ (MCH = 59.75 ± 18.07 pg). The variation of the MCHC index showed a similar behavior to the MCH, with an increase until *t*_21_ and a reduction at *t*_28_, in the exposed group (ANOVA: 5.44 *p* < 0.001) (Figure 2f,e; Table S1). The erythroblast count (Erb) did not vary between groups or throughout the experiment (ANOVA: 2.08 *p* > 0.05) (Figure 3a); a similar result was observed in the leukocyte count or white blood cell (WBC) (ANOVA: 0.6 *p* > 0.05) (Figure 3b). On the other hand, the thrombocyte count (Trb) varied significantly throughout the exposure (KW: 49.67 *p* < 0.001). An increase in the thrombocyte count was observed between *t*_0_ and *t*_7_, followed by a reduction, which remained until the end of the experiment (*t*_28_), whose pattern occurred in both experimental groups (Figure 3c).

### 2.4 Biomarkers of Genotoxicity

#### 2.4.1 Micronucleus Test and Nuclear Anomalies

The frequencies of MN and ENA were recorded at *t*_0_ and *t*_28_. In the samples collected at *t*_0_, there was no variation between the groups; however, at the end of the experiment (*t*_28_), the exposed group showed an increase in the frequency of the following biomarkers: MN %, Bud %, Bl % and Not % (Table 2).

**Table 2:** Frequency of Micronuclei (MN) and Erythrocyte Nuclear Abnormalities (ENA) in Erythrocytes of Juvenile Tilapia Exposed to a Diet Enriched with Methylmercury (0.5 mg kg^–1^)

	*t*_0_		*t*_28_	
	CG	EG	CG	EG
% MN	0	0	0.083 ± 0.11	0.2 ± 0.14***
% Bn	0 ± 0.015	0	0 ± 0.09	0 ± 0.08
% Bud	0.025 ± 0.07	0.05 ± 0.04	0.4 ± 0.44	0.9 ± 0.35***
% Bl	0.05 ± 0.04	0.07 ± 0.08	0.15 ± 0.11	0.65 ± 0.43***
% Not	0 ± 0.04	0.025 ± 0.05	0.8 ± 1.00	1.18 ± 1.13***
% Lob	0 ± 0.1	0.05 ± 0.05	0.1 ± 0.25	0.65 ± 0.77

*** (highly significant difference).

#### 2.4.2 Comet Assay on Fish Erythrocytes

**Figure 4:**
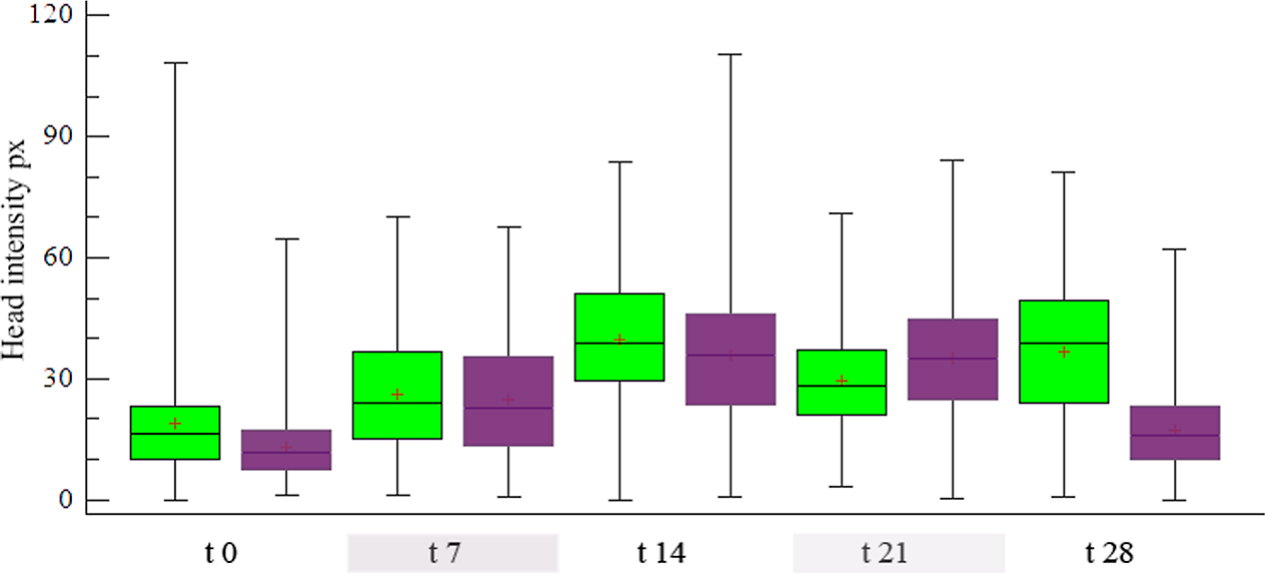
Comet head intensity in erythrocytes of juvenile tilapia over time exposed to methylmercury 0.5 mg kg-1. In the graph, the rectangular box shows the range of the data divided by a segment that indicates the median, the x represents the mean, and the vertical bar shows the dispersion of the data with the minimum and maximum values. Exposure time at 0, 7, 14, 21, and 28 days. CG (■(green)), EG (■(purple)).

DNA damage, assessed by intensity variation at the comet head, showed variation over time and between groups (KW: 2832.99 *p* < 0.001). From the beginning of the experiment (CG 0:16.30 ± 13.36 px; EG 0:11.70 ± 7.67 px; CG 7:24.16 ± 14.20 px; EG 7:22.85 ± 14.16 px; CG 14:38.74 ± 15.26 px; EG 14:35.86 ± 16.22 px) until the 21st day of exposure, an increase in the intensity of the head could be observed, although in the EG it was smaller than the CG, except on day 21, when the increase in the values of the exposed group was reached (CG 21 28.34 ± 12.66 px; EG 21:35.01 ± 15.26 px) and then decreased. at the end of the exposure bioassay (EG 28:15.98 ± 9.56 px) while the CG increased to 38.93 ± 16.90 px. On the other hand, it is worth noting that the EG had greater dispersion and atypical values, indicating individual variations in response to the toxicant (Figure 4).

### 2.5 Association between Variables

#### 2.5.1 Simple Linear Regression

In order to understand the magnitude and direction of the association between the different biomarkers evaluated in response to the daily consumption of food enriched with MeHg (0.5 mg kg^–1^) over 28 days. First, the linear regression test was performed to determine the dependence of each biomarker on blood Hg concentrations, finding that growth performance, the different hematimetric indices (MCH, MCV, MCHC) and Lob % and CA among the genotoxic biomarkers presented a relatively weak relationship, but nevertheless, among the variables that had a moderately strong positive association are Hct, Hgb, RBC, WBC, MN and the other ENAs, and the Erb count presented a relatively strong positive association; on the other hand, the Trb presented a moderately strong negative association (Table 3).

**Table 3:** Associations between THg Concentration in Blood and the Different Biomarkers Evaluated

	Hct	Hgb	RBC	Mn	Bud	Bl	Not	Bn	Erb	WBC	Trb
cc	0.74	0.73	0.72	0.72	0.78	0.84	0.80	0.71	0.92	0.60	–0.64
Β	846.46	130.03	26.87	4.85	10.80	30.29	58.27	4.75	3.41	861916.0	–2715.8
*R*^2^ (%)	55.96	53.14	51.59	51.87	60.37	70.80	64.23	49.73	84.49	36.45	41.35

#### 2.5.2 Spearman’s Correlation Coefficient

From biomarkers with moderate and relatively strong dependence on the accumulation of mercury in blood, and now with the purpose of determining the degree of association between two variables, the Spearman correlation coefficient was applied, which is a way of determining the fluctuation, oscillation or covariance existing between two parameters, determining the similarity in behavior, cause and consequences of one on the other. In general, a high correlation between biomarkers indicates that they have similar origins and/or analogous metabolic behaviors.

Eight sets of positive associations were established between Hgb and RBC, Erb and WBC; RBC with Erb and WBC, as well as between Erb and WBC; Hct was related to RBC and %Bn. On the other hand, relationships were found between the different genotoxicity biomarkers, MN with Bn %, Bud %, Bl %, and Not %; between Bn % and Bud % with Bl % and Not %; as well as a relationship between %Bl and Not %. In addition to these associations, negative relationships were also found between Trb and MN, Bud %, Bl %, and Not % (Tab. S2).

#### 2.5.3 Principal Component Analysis (PCA)

**Figure 5:**
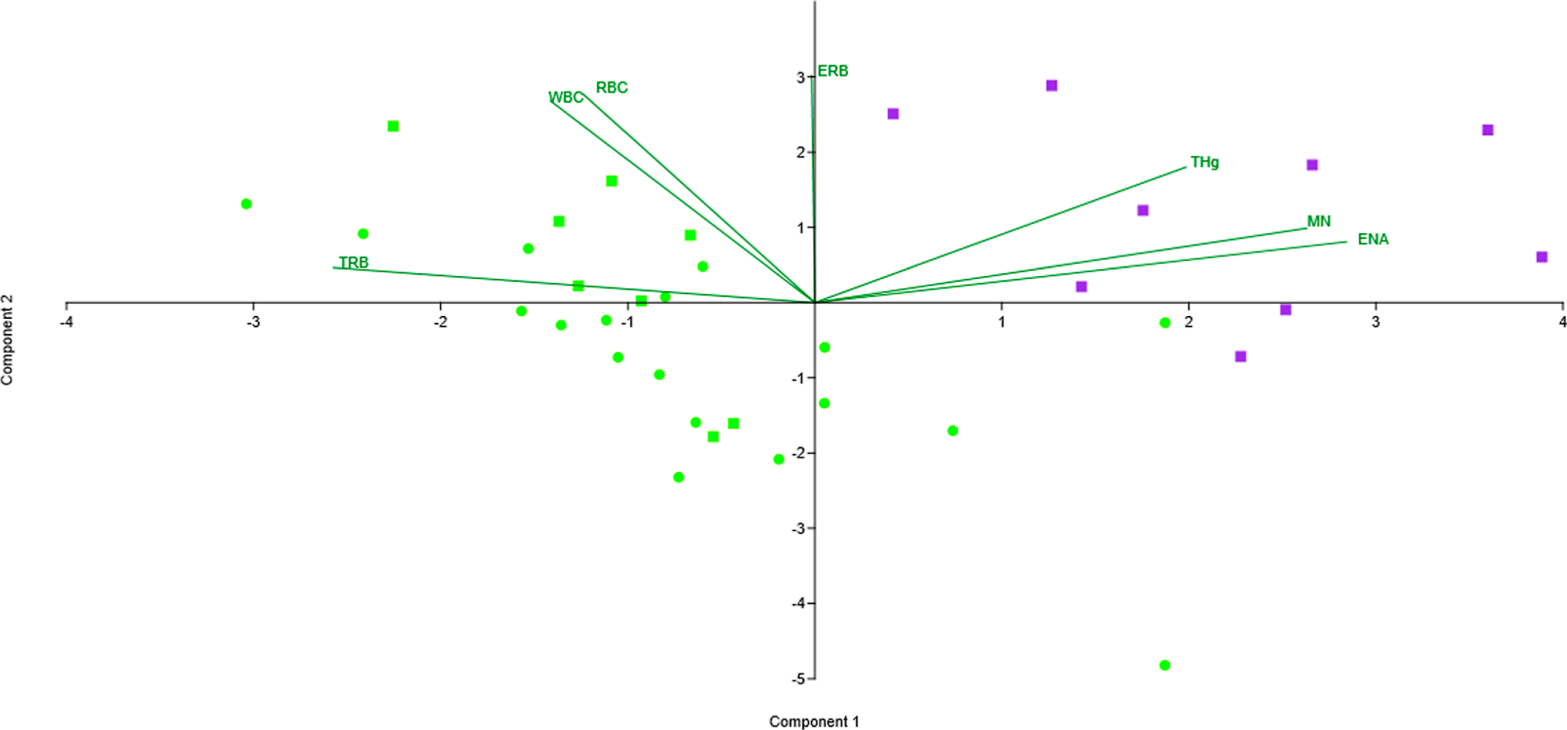
Principal component analysis between variables associated with mercury bioaccumulation in the blood of Tilapia Juveniles up to 28 days of exposure. CG (●(green)), EG *t*_0_ (■(green)), EG *t*_28_ (■(purple)).

The PCA ordering test revealed that the first two components explain 78.36% of the data variability; the first component presents the largest variation, explaining 41.76% including positive correlations between THg, ENA, MN and negative with Trb; the second component represented by positive correlations between THg with Erb, RBC and WBC, and an explanatory variation of 36.60%. In Figure 5 (Table S3) it can be detailed how the organisms exposed on the 28th day were spatially grouped and differentiated by THg vector, contrasting with CG and EG at the experiment beginning (*t*_0_), where no influence by MeHg could be detected.

## 3 Discussion

Mercury is considered one of the most toxic trace metals due to its ability to bioaccumulate and biomagnify in the food chain. The consumption of fish contaminated with mercury represents a serious public health problem in regions where fish is the main source of protein in the population’s diet, which is the case in several regions of the Amazon basin.^12,15,33−37^ In this study, using a model of daily consumption of food contaminated with methylmercury (0.5 mg kg^–1^) for 28 days, which is the maximum concentration established by the FDA, EPA (for the consumption of small fish, piscivorous or not) (UNEP; WHO, 2008), the possible effects on growth performance, hematological parameters and genotoxicity were evaluated.

Mercury exposure can affect the growth and general health of fish, the effect of which can be modulated by the time and dose of exposure.^19,38^ In addition, it is important to consider additive effects due to bioaccumulation and/or biomagnification processes, which depend on the species or trophic level of the fish.^19,39−41^ In this study, the growth of tilapia juveniles was not affected after 28 days of exposure to a diet supplemented with MeHg (0.5 mg kg^–1^). However, a longer exposure (60 days) using the same concentration was sufficient to decrease the weight and length of the animals.^19^ A similar result was obtained in juvenile of Stizostedion vitreum exposed for 6 months to MeHg (1.0 mg kg^–1^)^38^ and in the work carried out by Pratrap (2016),^24^ HgI concentrations of 0.01, 0.05, and 0.10 mg·L^–1^ were used for up to 35 days of exposure in water, affecting appetite, absorption rate and growth of tilapia with greater accumulation of Hg in gills than in muscle.

At the beginning of the experiment, the mercury concentrations of the fish from both groups showed values between 0.004 and 0.01 mg kg^–1^, which although low, indicate a possible source of entry of the metal into the culture medium from which the organisms were acquired, corroborating natural sources in the soil, which are made available by anthropic activities, which in this particular case are possibly: deforestation, removal of land for the creation of culture ponds, in addition to the possible influences of leaching processes in rainy seasons; as reported by Oestreicher et al. (2017).^8^

Mercury bioaccumulation was observed in blood and muscle samples. The body accumulation rate of 41% observed in tilapia juveniles can be explained by the 28 day exposure time. Friedmann et al. (1996)^38^ observed body accumulation rates at the different concentrations tested of 68 and 88% after 6 months of exposure. Berntssen et al. (2004)^40^ reported an accumulation of 83% in Atlantic salmon exposed for 4 months, with the highest accumulation rate occurring in the blood, followed by intestine, kidney and muscle. Studies of the kinetic distribution of MeHg via food in *Salvelinus alpinus* showed that the transfer of mercury between the intestine and the blood is slow, taking 27 days for 95% transfer, while for peripheral organs it lasts around 48 days.^42^ In *Ictalurus punctatus*, MeHg uptake in the intestinal epithelia occurs through passive and active processes, and/or through the energy-dependent neutral amino acid transporter, depending on the MeHg complexes.^43^ It is coherent to hypothesize that a longer exposure of tilapia fingerlings, under a dose of 0.5 mg kg^–1^, can induce a higher percentage of body accumulation and a reduction in growth performance and feeding habits.

Other research has shown that under long-term dietary exposure to MeHg, the bioaccumulation pattern tends to decrease the faster the fish grow. When exposed to high concentrations (e.g., 13.5 mg kg^–1^), mercury presents an accelerated bioaccumulation rate of up to 21 days and then decreases, affecting muscle fibers, suggesting adverse effects on the respiratory chain and mitochondrial distribution.^44^ In Nile tilapia exposed to concentrations of 0.5 to 2 mg kg^–1^ of MeHg for 30 days of exposure, the following were observed: greater aggressiveness, decreased swimming capacity by decreasing the activity of acetylcholinesterase and the immune system, reflecting in increases in molecules involved in the redox cycle (lysozyme, NO, SOD, MDA), affecting GSH concentrations implying greater oxidative stress.^39^

In humans, studies have demonstrated an association between mercury bioaccumulation and the consumption of fish with high health risks in riverside locations and/or near mining activities, as well as with cytogenetic damage in lymphocytes.^12,33,37,45−47^ Therefore, when one of the main sources of entry into the body is through food ingestion, the digestive system plays a crucial role, presenting high rates of intestinal absorption to then be transferred to the bloodstream and stored and/or purified in the different organs.^40,43^

Hematological parameters are frequently used in ecotoxicity studies, as one of the first tools to assess general health in fish.^7,20,22,48−52^ The hemogram, a set of biomarkers analyzed to determine the different components of the blood, divided into three categories: erythrogram, leukogram and thrombogram. In the erythrogram we can evaluate the erythrocyte count (RBC), the hemoglobin content in the blood (Hgb), the hematocrit percentage (Hct), as well as the hematimetric indices helping us to determine the size and color of the red blood cells present in the body’s blood and informing their condition, being able to identify and/or classify anemias; Among these indexes we find the mean corpuscular volume (MCV) indicating the size of the red blood cells; the mean corpuscular hemoglobin (MCH) and mean corpuscular hemoglobin concentration (MCHC) used as indicators of the coloration of the red blood cells as hypochromic and normochromic. The leukogram can be evaluated with the relative and/or absolute count of the different leukocytes and the thrombogram implies the count of thrombocytes in blood.^51−53^

The effects of heavy metal toxicity such as MeHg on red blood cells can manifest as plasma membrane instability, increased oxidative stress, altered antioxidant response, lipoperoxidation, variation in hematimetric indices, presence of anemia, increased changes in cellular and nuclear morphology, damage to genomic material, triggering necrosis and cell death.^19,27,48,50^ The hematological biomarkers evaluated in this study demonstrated that the erythrogram indices (RBC, Hct and MCV) and leukocyte, erythroblast and thrombocyte counts were not altered by the consumption of mercury-contaminated feed (0.5 mg kg^–1^) over time (up to 28 days). However, even in the absence of significant differences between the groups, the mean RBC was always higher in the exposed group, possibly associated with a subtle increase in erythropoiesis manifested with a slight increase in Erb counts. On the other hand, the variations in Hgb, MCHC and Trb appear to be associated with physiological behaviors. In the EG it was possible to detail that the Hgb, MCHC and Trb indexes showed a greater dispersion of data with a tendency for a reduction in values. The MCH index clearly showed a response to the negative effect of mercury from 21 days of exposure, reflecting a hypochromic anemia in the exposed organisms when compared with the CG.

Some authors have reported that the different parameters that make up fish hematology could be influenced by environmental and/or seasonal factors such as temperature, pH, population density, culture modality, as well as physiological growth factors, between species and even by stress associated with the blood sample collection procedure.^22,49,54^ Berntssen et al. (2016)^20^ reported seasonal variations with increases in erythrocytes and hematocrits during winter and summer, and among leukocyte differentiation, they presented a greater number of lymphocytes and neutrophils specific to the species. On the other hand, studies by Dal’Bó et al. (2015)^7^ demonstrated variations between species in hematological parameters and, in the case of tilapia, presented low amplitude in the values of Hgb, Hct, RBC and MCHC.

Short-term exposures (e.g., up to 28 days) with low concentration doses of MeHg are sufficient and appear to have an effect on the variation of hematological indices, as observed in the present study with tilapia juveniles. Seriani et al. (2015)^50^ observed a significant reduction in RBC and WBC indices between 3 and 14 days in tilapia exposed to 0.08 mg L^–1^ of HgCl_2_ diluted in aquarium water and Pratap (2016)^24^ report an increase of MCV but decreased of RBC, Hgb, PCV (Hct), MCHC and MCV. Longer exposures, regardless of the concentration administered, resulted in severe changes in hematological parameters, including RBC, Hgb and Hct indices.^19,40^

Additionally, genotoxicity parameters in juvenile tilapia were also affected by exposure to MeHg. Erytrocytic nuclear alterations (ENA) and micronuclei (MN) reached a maximum frequency of 1.18% (Not) in the exposed group, followed by Bud > Bl > Lob > Mn > Bn, with significant differences at the end of the experiment, which may demonstrate a clear increase in the formation of different alterations in response to daily ingestion of food contaminated with MeHg (0.5 mg kg^–1^).

The formation of micronuclei has been explained by events of missegregation of an entire chromosome or a fragment thereof during mitosis, being excluded from the nucleus in the daughter cell.^55^ Such events can be stimulated by a variety of causes, including: genetic defects in proteins involved in mitosis and its checkpoints,^56^ high exposure to chemical genotoxins and endogenous ones generated by metabolic stress processes, deficiency of micronutrients essential for DNA replication and repair.^26,56,57^ Morphonuclear alterations have been hypothetically considered as prior events until the formation of MN.^56−58^ Cytogenotoxicity studies evaluating the frequencies of morphonuclear alterations with the blockade of cytokinesis with cytochalasin B (Cyt-B) have shown that after cell division, these alterations tend to manifest in the nucleus of daughter cells or the presence of MN in them, and in addition, it is suggested that Bud and Lob are the product of broken nuclear bridges.^26,57,59^ The increase in the frequency of MN and ENAs has been related to developmental defects, cancer, accelerated aging in humans. In addition, there is evidence that the DNA present in MN can be recognized by the innate immune system, triggering inflammatory processes through cyclic GMP-AMP synthase (cGAS) and activating the stimulator of interferon genes (STING).^57^

Several authors have reported a clear association between a variety of mutagenic agents, such as monocrotophos insecticides (MCP), γ-radiation, environments with polluted water, and the increased frequency of MN and ENA.^26,27,60^ The effect of exposure to mercury leading to an increase in the frequency of MN and ENAs has been previously demonstrated, both in experimental bioassays,^50^ and in environments where high concentrations of mercury were found in fish muscle compared to reference sites.^18,30,61^ On the other hand, some studies of exposure to various toxins such as pesticides or even MeHg did not demonstrate an effect of exposure on the frequency of MN and ENAs or dose–response relationship, which possibly occurs due to the chemical and kinetic nature of the test substance,^31^ or in the case of MeHg, due to high concentrations in a short exposure time.^62^

The genotoxic effect of MeHg on juvenile tilapia was also manifested by increased DNA damage, as evidenced by the comet assay. There was a clear decrease in comet head intensity in the exposed group, suggesting cumulative DNA damage during the exposure period. In EG, the high dispersion of data and greater number of atypical values over time could indicate that some of the organisms are more resistant or susceptible to MeHg damage, responding with greater or lesser magnitude of repair mechanisms. Notably, up to the 21st day, a positive compensatory response in the repair of damage was still observed, which decreased drastically until the end of the experiment on the 28th day, corroborating the changes reflected in the increase in the frequencies of MN and ENAs, and in the hematological biomarkers MCH and Hgb, leading to the hypochromic condition at the end of the exposure time. The effects of damage to genetic material found in the present study could be compared to the data reported by Fatima et al. (2015)^28^ and Hussain et al. (2018),^30^ which showed an increase in genotoxic damage in fish from rivers contaminated by heavy metals including mercury.

The associations between Hg bioaccumulation and the different hematological and genotoxic biomarkers evaluated in this food exposure bioassay demonstrate that up to 28 days the concentration used does not directly influence behavior or variation in growth performance, but affects hematological and genotoxic biomarkers. The bioaccumulation process demonstrated in this study influenced the moderate and relatively strong positive associations with most of the parameters evaluated, except for thrombocytes, which had negative associations. In addition to these associations with MeHg, significant associations were demonstrated between the various hematological components and between them and Hgb; between Hct and RBC and Bn; as well as between MN and ENAs and relationships between the various ENAs. Notably, the frequency of ENAs and MN increases with mercury bioaccumulation, which in turn influences the increase in erythroblasts. It was expected that there would be associations between Erb with MN and ENAs, however, there was no correlation. A possible explanation would be that the bioaccumulation of Hg observed in the blood affected erythropoiesis activation mechanisms, manifesting itself in the increase of Erb associated with WBC and RBC, possibly as a vital response mechanism to the damage caused to cell membranes and genomic material, which could be proven by the notable increase in the frequencies of hematological (Hct, Hgb, RBC) and genotoxic (Bl, Bud, Not, MN) parameters.

According to the results observed in the MeHg dietary exposure test, we can suggest a hypothesis about the mechanism of origin of the morphonuclear alterations. Initially, the process of bioaccumulation of mercury in the blood directly influences the production of Erb, reflecting in the increase of Hct, possibly as a compensatory mechanism to the increase in the frequency of nuclear anomalies (Bud, Bl as previous stages), which during the following mitosis process can lead to the formation of MN in one of the daughter cells; and in the case of Not, in a smaller proportion, it could even lead to the formation of binucleated cells (represented with a lower frequency of Bn at the end of the exposure), considering that the cell membrane of the red blood cells did not show signs of future cytokinesis, understanding the principle that some change occurred in the process of cell division; according to the works done by Anbumani and Mohankumar (2011),^26^ Fenech (2020),^57^ Shimizu (2000),^58^ Shimizu et al. (1998)^56^ and Kwon et al. (2020)^63^ where they reported the study of MN and ENAs with the blockade of cytokinesis. On the other hand, the negative influence or association on thrombocyte counts with the bioaccumulation of MeHg over time may be due to the thiol–disulfide groups in the intrinsic proteins in the cellular membrane that can form bonds with Hg, altering the cellular redox state as well as their function and integrity^64−66^ (Tables 3, S5, Figure 5, Table S6).

## 4 Conclusions

Juvenile tilapia exposed to MeHg through dietary intake for 28 days exhibited significant mercury bioaccumulation and alterations in hematological and genotoxic parameters. as well as the negative association with Trb, were a direct consequence of MeHg exposure. Hematological parameters such as Erb, RBC, and WBC likely represent secondary and/or compensatory effects of metabolic responses to mercury exposure. The greatest variations between the control group and the exposed group occurred between days 21 and 28, a period in which the organisms defense response may have broken down to compensate for the constant entry and accumulation of the toxic substance in the bloodstream. Monitoring genotoxicity associated with mercury exposure is important to ensure adequate management of fish health and food security for riverside populations in the Amazon region.

## 5 Methods

### 5.1 Substance and Organism Tests

To induce exposure to Hg via food intake was provided with a commercial feed (Supravit Juvenil 46, 1.7 mm), which was impregnated with a methylmercury chloride solution (CH_3_HgCl_2_), according to procedures adapted from Alam et al. (2021)^19^ and Berntssen et al. (2004).^40^ From a stock solution of CH_3_HgCl_2_ 1 mg mL^–1^ of absolute ethanol, the desired concentration (Hg 0.5 mg kg^–1^ of feed) was prepared and completed to 10 mL with absolute ethanol. A batch with 130 g of feed was separated and placed in a tray to be sprayed with the methylmercury solution with constant stirring to ensure impregnation of the grains. This procedure was carried out inside the exhaust hood and allowed to evaporate for 24 h (h). A sample of the impregnated food grains was examined with a Direct Mercury Analyzer DMA-80 to quantify the incorporated mercury content. The final mercury concentration in food was calculated with the desired exposure concentration, in triplicate, and then aliquots were made in quantities corresponding to daily doses equivalent to 2% of the body mass of each fish. The aliquots for the daily meal were stored in airtight bags and kept in the freezer at −20 °C.

The test organisms, 20 healthy juvenile tilapias (*Oreochromis* sp.) were obtained commercially from a fish farming company located on Highway PA-457, Alter do Chão, Santarém-PA. The fish acclimated to laboratory conditions for 15 days, with a photoperiod of 12 h light/12 h dark, with constant aeration; pH 6.96 ± 0.2; EC 0.13 ± 0.02 μS cm-1; TDS 0.06 ± 0.01; temperature of 25.05 ± 1 °C, and were monitored daily with a multiparameter probe meter (Hanna instruments Inc., HI 9811-5, Woonsocket, USA).

### 5.2 Experimental Design

The fish were divided into two experimental groups (*n* = 10/group, weight: 15.57 ± 5.06 g, CT: 9.99 ± 1.06 cm); the exposed group (EG) received food added with methylmercury (0.5 mg kg^–1^ diet) and the control group (CG) received the mercury-free Supravit Juvenil 46 food, at the same daily dosage equivalent to 2% of body mass (weekly adjusted). The fish were kept exposed to mercury for 28 days in glass aquariums with a capacity of 20 L with a density of 5 fish per aquarium in an approximate ratio of 1 g of fish per L of water, which received daily siphoning aspiration to clean them and avoid contamination by ingestion of feces, and every 2 days 50% of the water volume was replaced^19,39−41^ (Figure S1). At the end of the experiment, photographs, size and weight data of the fish were taken for analysis of growth performance; the organisms were cryo-anesthetized/euthanized and immediately submitted for extraction of blood and target tissues for the research, according to a procedure approved by the Ethics Committee for Research with Animals of the Federal University of Western Pará (CEUA no. 0520230254) (Figure S2) and SISBIO protocol no. 86173-1 (Figure S3). Approximately 0.5 g of white muscle was removed from each fish, placed in microtubes and stored at −20 °C for later analysis of the total mercury (THg) concentration.

### 5.3 Growth Performance Analysis

To evaluate this parameter, the body weights of the fish were determined from the beginning of the experiment until the end of the study (28 days) at weekly intervals, to determine the fish’s feed intake.^19^ Determining at the end of the study (28 days) the final body weight. Weight gain (WG, g), daily weight gain (DWG, g day^–1^), specific growth rate (SGR) and feed conversion ratio (FCR) were determined using the following formulas
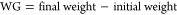

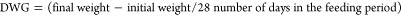

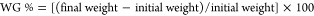

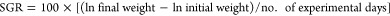

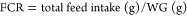


### 5.4 Determination of Total Mercury

Method 7473 recommended by the United States Environmental Protection Agency (EPA) was used to determine Total Hg (THg), which consists of directly determining it (inorganic and organic) without sample preparation. The tissue samples were previously thawed and weighed, and analyzed in duplicate by atomic absorption spectrophotometry using the DMA-80 apparatus (Milestone Srl, Sorisole, Italy), whose detection limit is 0.0015 ng.^67^ Part of blood samples (0.05–0.10 g) collected throughout the experiment every 7 days were analyzed and grouped into pools according to time (*t*_0_, *t*_7_, *t*_14_, *t*_21_, *t*_28_ days) and the control and exposed group; muscle tissue samples (0.10–0.30 g) were analyzed at the end of the experiment (28 days). Mercury concentration was expressed as mg kg^–1^.

### 5.5 Body Accumulation Rate

From the Hg concentrations in the muscles of the fish in both groups (CG and EG) it was possible to calculate the amount of accumulated metal using the following formula
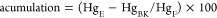
where Hg_E_ is the average Hg content/exposed fish; Hg_BK_ is the average Hg content/control fish and Hg_F_ is the amount of THg consumed by fish over time (28 days).^38^

### 5.6 Hematological Biomarkers

The juveniles were sampled weekly according to times (*t*_0_, *t*_7_, *t*_14_, *t*_21_, *t*_28_ days), between 0.2 and 0.3 mL of blood was collected from a caudal vein with heparinized syringe (heparin solution 100 IU).^53^ The hemoglobin (Hgb) and thrombocyte (Trb) counts were processed on the same day of blood collection using an automated method following protocols adapted from Romão et al., (2006)^54^ and Rodrigues et al., (2010).^68^ The erythrocyte or red blood cell (RBC) counts was performed immediately diluting in a physiologic solution (0.65%) with a Neubauer’s hemocytometer. Leukocyte or white blood cell (WBC) counts was performed up to a month later by the indirect method manually from the May Grünwald Giemsa Wright (MGGW) stained smears (permanently monted) by the erythrocyte/leucocyte ratio.^53^ The hematocrit (Hct %) was obtained by the microhematocrit technique with centrifugation at 14,000*g* for 5 min. Mean corpuscular volume (MCV) was calculated as follows: MCV = [(Hematocrit) × 100]/(total red blood cell count). Mean corpuscular hemoglobin (MCH) was calculated as follows MCH = Hgb × 10/RBC. Mean corpuscular hemoglobin concentration (MCHC) was calculated as described.^51,53,54^

### 5.7 Biomarkers of Genotoxicity

#### 5.7.1 Micronucleus (MN) and Erythrocytic Nuclear Anomalies (ENAs) Testing of Fish

Blood smears were made in duplicate for each individual and allowed to dry at room temperature for 24 h. The slides were stained with MGGW stain for 3 min and then diluted in phosphate buffer pH 6.8 for 11 min, then washed with running water and dried at room temperature. The slides were analyzed under an optical microscope under 1000× magnification, with 2000 erythrocytes counted per individual.^53^ To identify the micronucleus, it was considered as a nonrefracting round structure, with approximately 1/10–1/30 of the area of the nucleus and separated from the erythrocyte nucleus.^55^ The ENAs were also recorded on the same slides prepared for micronucleus analysis, being classified into categories according to Carrasco et al. (1990)^31^ and Anbumani & Mohankumar (2011).^26^ The frequency of micronuclei and ENAs were calculated using the following formula
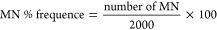


#### 5.7.2 Comet Assay on Fish Erythrocytes (CA)

The alkaline assay was used as described by Silva, (2007)^69^ with modifications in the staining steps. The cells adhered to the agarose layer were incubated in lysis buffer (2.5 M NaCl; 100 mM EDTA; 10 mM TRIS, added with 1% Triton X-100 e 10% DMSO immediately before lysis procedure) for at least 3 h, under refrigeration. The electrophoresis conditions were 25 V, 300 mA, 100 W for 20 min, with the electrophoresis tank cooled to avoid overheating of the buffer. After electrophoresis, the slides were washed 3x with neutralizing buffer (0.4 M Tris Hydroxymethane-HCl) for 5 min, washed 2x with ice-cold distilled water, and fixed with ice-cold ethanol for 10 min, and dried for 2 h in an oven at 37 °C. The fixed slides were stored in the refrigerator until subsequent staining with 0.002 mg mL^–1^ DAPI (Diamidino phenylindole) and H-1000 fluorescence mounting medium (Vectashield, Vector Laboratories Inc. Burlingame, CA)^70^ and analysis on a Nikon Eclipse Ci-S fluorescence microscope (Nikon Corporation, Tokyo, Japan) under 100× magnification.

For the analysis, 5 photographs of each slide were captured, with approximately 100 cells per slide. The evaluation of cellular damage was performed semiautomatically, with the aid of the free software ImageJ and Open Comet plugin. The PNG images were processed and each comet was manually checked, excluding invalid or outliers. The comet head intensity parameter (px) was used to perform cell damage calculations, understanding that the lower the intensity values, the greater the damage to the genetic material.^71^

### 5.8 Statistical Analysis

The normality of the data of the different variables was assessed using the Kolgomorov–Smirnov test. Statistical analyses of the differences between groups over the exposure time were performed using the one-way ANOVA (blood Hg, WG, DWG, WG %, SGR, FCR, Hct, Hgb, RBC, MCH, MCHC, WBC) and nonparametric Kruskal–Wallis’s test (muscle Hg, Trb, MCV, MN and ENA, CA) with a posteriori correction using Tukey’s HSD multiple comparison test. The association between blood mercury concentration in the exposed group and the various biomarkers evaluated was first performed using linear regression for each variable, followed by Spearman correlation analysis To evaluate the association of Hg bioaccumulation and multiple biomarkers we done a principal component analysis (PCA) through the measurements observed at the beginning (*t*_0_) and at the end of the experiment (*t*_28_); the ENA values were represented by the sum of the frequencies of Bn + Bud + Bl + Not, and considering the variables that best explain the variation (THg, RBC, Erb, WBC, Trb, MN and ENA), standardized data matrix for removing the scale effect of measurements (x-mean)/SD and thus be able to graph the analysis; using the statistical package STATGRAPHICS 16.0.

## Supporting Information Available

Summary table of biomarkers in juveniles Tilapia exposed to MeHg 0.5 mg kg^–1^; Relationship between variables associated with THg in juveniles exposed to MeHg 0.5 mg kg^–1^; Relative significance of variances of the variables associated with mercury bioaccumulation in blood of juvenile Tilapia up to 28 days of exposure; Juvenile tilapia in a bioassay of exposure to methylmercury by food intake; Certificate from the Animal Research Ethics Committee of the Federal University of Western Pará (UFOPA); Protocol for collecting organisms for research purposes SISBIO no. 86173-1 (PDF)

## References

[ref1] Naylor R. L.; Goldburg R. J.; Primavera J. H.; Kautsky N.; Beveridge M. C. M.; Clay J.; Folke C.; Lubchenco J.; Mooney H.; Troell M. Effect of Aquaculture on World Fish Supplies. Nature 2000, 405 (6790), 1017–1024. 10.1038/35016500. 10890435

[ref2] Neves P. R.; Ribeiro R. P.; Vargas L.; Natali M. R. M.; Maehana K. R.; Marengoni N. G. Evaluation of the Performance of Two Strains of Nile Tilapia (Oreochromis Niloticus) in Mixed Raising Systems. Braz. Arch. Biol. Technol. 2008, 51 (3), 531–538. 10.1590/S1516-89132008000300012.

[ref3] Sastraprawira S. M.; Abd. Razak I. H.; Shahimi S.; Pati S.; Edinur H. A.; John A. B.; Ahmad A.; Kumaran J. V.; Martin M. B.; Chong J. L.; Chowdhury A. J. K.; Nelson B. R. A Review on Introduced Cichla Spp. and Emerging Concerns. Heliyon 2020, 6 (11), e05370 10.1016/j.heliyon.2020.e05370. 33204875 PMC7648196

[ref4] Boletim-Da-Aquicultura-Em-Aguas-Da-Uniao-2013–2022-Site_compressed. https://www.gov.br/mpa/pt-br/Central_Conteudos/arquivos-docs-ppts/boletim-da-aquicultura-em-aguas-da-uniao-2013-2022-site_compressed.pdf (accessed 2025-01–08).

[ref5] Ferreira Brabo M.; Cristina Do Nascimento Matos S.; Helena Pamplona Façanha Serra R.; Gustavo Bezerra Costa B.; Abreu Vasconcelos Campelo D.; Crovatto Veras G. A tilapicultura no estado do Pará, Amazônia. Informações Econômicas 2020, 50, 1–11. 10.56468/1678-832X.eie1018.2020.

[ref6] Silva Y. Y.; Silva R. d. N.´ P. da.; Almeida E. G. d..; Vilarinho C. C. A piscicultura no território do sistema norte: Pará e Maranhão, 2021. 10.29223/PROD.TEC.ITV.DS.2021.36.Silva.

[ref7] Dal’Bó G. A.; Sampaio F. G.; Losekann M. E.; Queiroz J. F. D.; Luiz A. J. B.; Wolf V. H. G.; Gonçalves V. T.; Carra M. L. Hematological and Morphometric Blood Value of Four Cultured Species of Economically Important Tropical Foodfish. Neotrop. Ichthyol 2015, 13 (2), 439–446. 10.1590/1982-0224-20140115.

[ref8] Oestreicher J. S.; Lucotte M.; Moingt M.; Bélanger E. ´.; Rozon C.; Davidson R.; Mertens F.; Romaña C. A. Environmental and Anthropogenic Factors Influencing Mercury Dynamics During the Past Century in Floodplain Lakes of the Tapajós River, Brazilian Amazon. Arch. Environ. Contam. Toxicol. 2017, 72 (1), 11–30. 10.1007/s00244-016-0325-1. 27858105

[ref9] De Souza R. E.; Fontes M. P. F.; Tucci C. A. F.; Lima H. N.; Da Silva Ferreira M. Health Risk Assessment and Quality Reference Values of Potentially Toxic Elements in Soils of the Southwestern Amazonas State – Brazil. Sci. Total Environ. 2024, 912, 168937 10.1016/j.scitotenv.2023.168937. 38029983

[ref10] Telmer K.; Costa M.; Simões Angélica R.; Araujo E. S.; Maurice Y. The Source and Fate of Sediment and Mercury in the Tapajós River, Pará, Brazilian Amazon: Ground- and Space-Based Evidence. J. Environ. Manage. 2006, 81 (2), 101–113. 10.1016/j.jenvman.2005.09.027. 16824670

[ref11] Roulet M.; Lucotte M.; Farella N.; Serique G.; Coelho H.; Sousa Passos C. J.; de Jesus da Silva E.; Scavone de Andrade P.; Mergler D.; Guimarães J. R. D.; et al. Effects of Recent Human Colonization on the Presence of Mercury in Amazonian Ecosystems. Water, Air, Soil Pollut. 1999, 112, 297–313. 10.1023/a:1005073432015.

[ref12] Oliveira R. B. D.; Silva D. M. D.; Franco T. S. B. S.; Vasconcelos C. R. S.; Sousa D. J. D. A. D.; Sarrazin S. L. F.; Sakamoto M.; Bourdineaud J.-P. Fish Consumption Habits of Pregnant Women in Itaituba, Tapajós River Basin, Brazil: Risks of Mercury Contamination as Assessed by Measuring Total Mercury in Highly Consumed Piscivore Fish Species and in Hair of Pregnant Women. Arch. Ind. Hyg. Toxicol. 2022, 73 (2), 131–142. 10.2478/aiht-2022-73-3611. PMC9287832 35792767

[ref13] Cammilleri G.; Galluzzo F. G.; Fazio F.; Pulvirenti A.; Vella A.; Lo Dico G. M.; Macaluso A.; Ciaccio G.; Ferrantelli V. Mercury Detection in Benthic and Pelagic Fish Collected from Western Sicily (Southern Italy). Animals 2019, 9 (9), 594 10.3390/ani9090594. 31443421 PMC6769492

[ref14] Montaña C. G.; Liverpool E.; Taphorn D. C.; Schalk C. M. The Cost of Gold: Mercury Contamination of Fishes in a Neotropical River Food Web. Neotrop. Ichthyol 2021, 19 (3), e200155 10.1590/1982-0224-2020-0155.

[ref15] Castilhos Z.; Rodrigues-Filho S.; Cesar R.; Rodrigues A. P.; Villas-Bôas R.; De Jesus I.; Lima M.; Faial K.; Miranda A.; Brabo E.; Beinhoff C.; Santos E. Human Exposure and Risk Assessment Associated with Mercury Contamination in Artisanal Gold Mining Areas in the Brazilian Amazon. Environ. Sci. Pollut. Res. 2015, 22 (15), 11255–11264. 10.1007/s11356-015-4340-y. 25797016

[ref16] Basta P. C.; Viana P. V. D. S.; Vasconcellos A. C. S. D.; Périssé A. R. S.; Hofer C. B.; Paiva N. S.; Kempton J. W.; Ciampi De Andrade D.; Oliveira R. A. A. D.; Achatz R. W.; Perini J. A.; Meneses H. D. N. D. M.; Hallwass G.; Lima M. D. O.; Jesus I. M. D.; Santos C. C. R. D.; Hacon S. D. S. Mercury Exposure in Munduruku Indigenous Communities from Brazilian Amazon: Methodological Background and an Overview of the Principal Results. Int. J. Environ. Res. Public Health 2021, 18 (17), 9222 10.3390/ijerph18179222. 34501811 PMC8430525

[ref17] Guimarães K. L. A.; do Nascimento Andrade S. J.; Liscano-Carreño A. A.; de Oliveira R. B.; Rodrigues L. R. R. Systematic Review and Spatiotemporal Assessment of Mercury Concentration in Fish from the Tapajós River Basin: Implications for Environmental and Human Health. ACS Environ. Au 2025, 5, 86 10.1021/acsenvironau.4c00053. 39830718 PMC11741060

[ref18] Porto J. I. R.; Araujo C. S. O.; Feldberg E. Mutagenic Effects of Mercury Pollution as Revealed by Micronucleus Test on Three Amazonian Fish Species. Environ. Res. 2005, 97 (3), 287–292. 10.1016/j.envres.2004.04.006. 15589237

[ref19] Alam R. T. M.; Abu Zeid E. H.; Khalifa B. A.; Arisha A. H.; Reda R. M. Dietary Exposure to Methyl Mercury Chloride Induces Alterations in Hematology, Biochemical Parameters, and mRNA Expression of Antioxidant Enzymes and Metallothionein in Nile Tilapia. Environ. Sci. Pollut. Res. 2021, 28 (24), 31391–31402. 10.1007/s11356-021-13014-5. 33606169

[ref20] Azevedo T. M. P. D.; Albanati R.; Guerra-Santos B.; Pinto L.; Lira A.; Medeiros; Ayres M. Valores de referência dos parâmetros hematológicos de Oreochromis niloticus (Linaeus 1758) cultivados em tanques-rede em Paulo Afonso, no Estado da Bahia, Brasil. Braz. J. Aquat. Sci. Technol. 2016, 20 (2), 63–74. 10.14210/bjast.v20n2.4588.

[ref21] De Jesus T. B.; De Carvalho C. E. V. Utilização de biomarcadores em peixes como ferramenta para avaliação de contaminação ambiental por mercúrio (Hg). Oecol. Austr. 2008, 12 (04), 680–693. 10.4257/oeco.2008.1204.07.

[ref22] Tavares-Dias M.; Tenani R. A.; Gioli L. D.; Faustino C. D. Características hematológicas de teleósteos brasileiros: II. Parâmetros sangüíneos do Piaractus mesopotamicus Holmberg (Osteichthyes, Characidae) em policultivo intensivo. Rev. Bras. Zool. 1999, 16 (2), 423–431. 10.1590/S0101-81751999000200008.

[ref23] Shahjahan M.; Taslima K.; Rahman M. S.; Al-Emran M.; Alam S. I.; Faggio C. Effects of Heavy Metals on Fish Physiology – A Review. Chemosphere 2022, 300, 134519 10.1016/j.chemosphere.2022.134519. 35398071

[ref24] Pratap H. B. Haematological Responses and Growth of African Freshwater Cichlids Oreochromis Niloticus Exposed to Ambient Inorganic Mercury. International Journal of Zoological Investigations 2016, 2 (1), 09–16.

[ref25] Sadiqul I. M.; Ferdous Z.; Nannu Md. T. A.; Mostakim G. M.; Rahman Md. K. Acute Exposure to a Quinalphos Containing Insecticide (Convoy) Causes Genetic Damage and Nuclear Changes in Peripheral Erythrocytes of Silver Barb, Barbonymus Gonionotus. Environ. Pollut. 2016, 219, 949–956. 10.1016/j.envpol.2016.09.066. 27667680

[ref26] Anbumani S.; Mohankumar M. N. Nuclear and Cytoplasmic Abnormalities in the Fish Catla Catla (Hamilton) Exposed to Chemicals and Ionizing Radiation. Res. J. Environ. Sci 2011, 5 (12), 867–877. 10.3923/rjes.2011.867.877.

[ref27] Carrola J.; Santos N.; Rocha M. J.; Fontainhas-Fernandes A.; Pardal M. A.; Monteiro R. A. F.; Rocha E. Frequency of Micronuclei and of Other Nuclear Abnormalities in Erythrocytes of the Grey Mullet from the Mondego, Douro and Ave Estuaries—Portugal. Environ. Sci. Pollut. Res. 2014, 21 (9), 6057–6068. 10.1007/s11356-014-2537-0. 24469770

[ref28] Fatima M.; Usmani N.; Firdaus F.; Zafeer M. F.; Ahmad S.; Akhtar K.; Dawar Husain S. M.; Ahmad M. H.; Anis E.; Mobarak Hossain M. *In Vivo* Induction of Antioxidant Response and Oxidative Stress Associated with Genotoxicity and Histopathological Alteration in Two Commercial Fish Species Due to Heavy Metals Exposure in Northern India (Kali) River. Comp. Biochem. Physiol., Part C:Toxicol. Pharmacol. 2015, 176–177, 17–30. 10.1016/j.cbpc.2015.07.004. 26191657

[ref29] Gajski G.; Žegura B.; Ladeira C.; Novak M.; Sramkova M.; Pourrut B.; Del Bo’ C.; Milić M.; Gutzkow K. B.; Costa S.; Dusinska M.; Brunborg G.; Collins A. The Comet Assay in Animal Models: From Bugs to Whales – (Part 2 Vertebrates). Mutat. Res., Rev. Mutat. Res. 2019, 781, 130–164. 10.1016/j.mrrev.2019.04.002. 31416573

[ref30] Hussain B.; Sultana T.; Sultana S.; Masoud M. S.; Ahmed Z.; Mahboob S. Fish Eco-Genotoxicology: Comet and Micronucleus Assay in Fish Erythrocytes as in Situ Biomarker of Freshwater Pollution. Saudi J. Biol. Sci. 2018, 25 (2), 393–398. 10.1016/j.sjbs.2017.11.048. 29472797 PMC5816008

[ref31] Carrasco K. R.; Tilbury K. L.; Myers M. S. Assessment of the Piscine Micronucleus Test as an in Situ Biological Indicator of Chemical Contaminant Effects. Can. J. Fish. Aquat. Sci. 1990, 47 (11), 2123–2136. 10.1139/f90-237.

[ref32] Goals Archive. The Global Goals. https://globalgoals.org/goals/ (accessed 10 10, 2024).

[ref33] Amorim M. I. M.; Mergler D.; Bahia M. O.; Dubeau H.; Miranda D.; Lebel J.; Burbano R. R.; Lucotte M. Cytogenetic Damage Related to Low Levels of Methyl Mercury Contamination in the Brazilian Amazon. An. Acad. Bras. Cienc. 2000, 72 (4), 497–507. 10.1590/S0001-37652000000400004. 11151017

[ref34] Hacon S.; Yokoo E.; Valente J.; Campos R. C.; da Silva V. A.; de Menezes A. C.; de Moraes L. P.; Ignotti E. Exposure to Mercury in Pregnant Women from Alta Floresta-Amazon Basin, Brazil. Environ. Res. 2000, 84 (3), 204–210. 10.1006/enrs.2000.4115. 11097793

[ref35] Lavoie R. A.; Jardine T. D.; Chumchal M. M.; Kidd K. A.; Campbell L. M. Biomagnification of Mercury in Aquatic Food Webs: A Worldwide Meta-Analysis. Environ. Sci. Technol. 2013, 47 (23), 13385–13394. 10.1021/es403103t. 24151937

[ref36] United Nations Environment Programme, World Health Organization. Guidance for Identifying Population at Risk from Mercury Exposure, 2008, p 176.

[ref37] Vasconcellos A. C. S. D.; Hallwass G.; Bezerra J. G.; Aciole A. N. S.; Meneses H. N. D. M.; Lima M. D. O.; Jesus I. M. D.; Hacon S. D. S.; Basta P. C. Health Risk Assessment of Mercury Exposure from Fish Consumption in Munduruku Indigenous Communities in the Brazilian Amazon. Int. J. Environ. Res. Public Health 2021, 18 (15), 7940 10.3390/ijerph18157940. 34360233 PMC8345402

[ref38] Friedmann A. S.; Watzin M. C.; Brinck-Johnsen T.; Leiter J. C. Low Levels of Dietary Methylmercury Inhibit Growth and Gonadal Development in Juvenile Walleye (Stizostedion Vitreum). Aquat. Toxicol. 1996, 35 (3–4), 265–278. 10.1016/0166-445X(96)00796-5.

[ref39] Abu Zeid E. H.; Khalifa B. A.; Said E. N.; Arisha A. H.; Reda R. M. Neurobehavioral and Immune-Toxic Impairments Induced by Organic Methyl Mercury Dietary Exposure in Nile Tilapia *Oreochromis Niloticus*. Aquat. Toxicol. 2021, 230, 105702 10.1016/j.aquatox.2020.105702. 33264694

[ref40] Berntssen M. h. g.; Hylland K.; Julshamn K.; Lundebye A.-K.; Waagbø R. Maximum Limits of Organic and Inorganic Mercury in Fish Feed. Aquacult. Nutr. 2004, 10 (2), 83–97. 10.1046/j.1365-2095.2003.00282.x.

[ref41] Wang R.; Wang W.-X. Diet-Specific Trophic Transfer of Mercury in Tilapia (Oreochromis Niloticus): Biodynamic Perspective. Environ. Pollut. 2018, 234, 288–296. 10.1016/j.envpol.2017.11.071. 29182973

[ref42] Oliveira Ribeiro C. A.; Rouleau C.; Pelletier E. ´.; Audet C.; Tjälve H. Distribution Kinetics of Dietary Methylmercury in the Arctic Charr (*Salvelinus Alpinus*). Environ. Sci. Technol. 1999, 33 (6), 902–907. 10.1021/es980242n.

[ref43] Leaner J. J.; Mason R. P. Methylmercury Accumulation and Fluxes across the Intestine of Channel Catfish, Ictalurus Punctatus. Comp. Biochem. Physiol., Part C:Toxicol. Pharmacol. 2002, 132 (2), 247–259. 10.1016/S1532-0456(02)00072-8. 12106901

[ref44] De Oliveira Ribeiro C. A.; Nathalie M.-D.; Gonzalez P.; Yannick D.; Jean-Paul B.; Boudou A.; Massabuau J. C. Effects of Dietary Methylmercury on Zebrafish Skeletal Muscle Fibres. Environ. Toxicol. Pharmacol. 2008, 25 (3), 304–309. 10.1016/j.etap.2007.10.033. 21783867

[ref45] De Flora S.; Bennicelli C.; Bagnasco M. Genotoxicity of Mercury Compounds. A Review. Mutat. Res., Rev. Genet. Toxicol. 1994, 317 (1), 57–79. 10.1016/0165-1110(94)90012-4. 7507573

[ref46] Malm O.; Branches F. J. P.; Akagi H.; Castro M. B.; Pfeiffer W. C.; Harada M.; Bastos W. R.; Kato H. Mercury and Methylmercury in Fish and Human Hair from the Tapajós River Basin, Brazil. Sci. Total Environ. 1995, 175 (2), 141–150. 10.1016/0048-9697(95)04910-X. 8560242

[ref47] Meneses H. D. N. D. M.; Oliveira-da-Costa M.; Basta P. C.; Morais C. G.; Pereira R. J. B.; De Souza S. M. S.; Hacon S. D. S. Mercury Contamination: A Growing Threat to Riverine and Urban Communities in the Brazilian Amazon. Int. J. Environ. Res. Public Health 2022, 19 (5), 2816 10.3390/ijerph19052816. 35270508 PMC8910171

[ref48] Farag M. R.; Alagawany M. Erythrocytes as a Biological Model for Screening of Xenobiotics Toxicity. Chem.-Biol. Interact. 2018, 279, 73–83. 10.1016/j.cbi.2017.11.007. 29128605

[ref49] Maceda-Veiga A.; Figuerola J.; Martínez-Silvestre A.; Viscor G.; Ferrari N.; Pacheco M. Inside the Redbox: Applications of Haematology in Wildlife Monitoring and Ecosystem Health Assessment. Sci. Total Environ. 2015, 514, 322–332. 10.1016/j.scitotenv.2015.02.004. 25668285

[ref50] Seriani R.; França J. G.; Lombardi J. V.; Brito J. M.; Ranzani-Paiva M. J. T. Hematological Changes and Cytogenotoxicity in the Tilapia Oreochromis Niloticus Caused by Sub-Chronic Exposures to Mercury and Selenium. Fish Physiol. Biochem. 2015, 41 (1), 311–322. 10.1007/s10695-014-9984-x. 25216806

[ref51] Tavares-Dias M.; Moraes F. R. d.. Hematologia de Peixes Teleósteos; Marcos Tavares-Dias, 2004.

[ref52] Witeska M.; Kondera E.; Ługowska K.; Bojarski B. Hematological Methods in Fish – Not Only for Beginners. Aquaculture 2022, 547, 737498 10.1016/j.aquaculture.2021.737498.

[ref53] Ranzani-Paiva M. J. T.; Pádua S. B. D.; Tavares-Dias M.; Egami M. I. Métodos para análise hematológica em peixes; EDUEM, 2013; . 10.7476/9788576286530.

[ref54] Romão S.; Donatti L.; Freitas M. O.; Teixeira J.; Kusma J. Blood Parameter Analysis and Morphological Alterations as Biomarkers on the Health of Hoplias Malabaricus and Geophagus Brasiliensis. Braz. Arch. Biol. Technol. 2006, 49 (3), 441–448. 10.1590/S1516-89132006000400012.

[ref55] Al-Sabti K.; Metcalfe C. D. Fish Micronuclei for Assessing Genotoxicity in Water. Mutat. Res., Genet. Toxicol. 1995, 343 (2), 121–135. 10.1016/0165-1218(95)90078-0. 7791806

[ref56] Shimizu N.; Itoh N.; Utiyama H.; Wahl G. M. Selective Entrapment of Extrachromosomally Amplified DNA by Nuclear Budding and Micronucleation during S Phase. J. Cell Biol. 1998, 140 (6), 1307–1320. 10.1083/jcb.140.6.1307. 9508765 PMC2132668

[ref57] Fenech M. Cytokinesis-Block Micronucleus Cytome Assay Evolution into a More Comprehensive Method to Measure Chromosomal Instability. Genes 2020, 11 (10), 1203 10.3390/genes11101203. 33076531 PMC7602810

[ref58] Shimizu N.; Shimura T.; Tanaka T. Selective Elimination of Acentric Double Minutes from Cancer Cells through the Extrusion of Micronuclei. Mutat. Res., Fundam. Mol. Mech. Mutagen. 2000, 448 (1), 81–90. 10.1016/S0027-5107(00)00003-8. 10751625

[ref59] Fenech M. Cytokinesis-Block Micronucleus Cytome Assay. Nat. Protoc. 2007, 2 (5), 1084–1104. 10.1038/nprot.2007.77. 17546000

[ref60] Silva A.; Nepomuceno J. C. Avaliação da frequência de micronúcleos em eritrócitos periféricos de mandi-amarelo (Pimelodus maculatus) do rio Paranaíba. Revista do Núcleo Interdisciplinar de Pesquisa e Extensão do UNIPAM 2010, 1 (7), 167.

[ref61] Cruz-Esquivel A. ´.; Díez S.; Marrugo-Negrete J. L. Genotoxicity Effects in Freshwater Fish Species Associated with Gold Mining Activities in Tropical Aquatic Ecosystems. Ecotoxicol. Environ. Saf. 2023, 253, 114670 10.1016/j.ecoenv.2023.114670. 36857922

[ref62] Rocha R. D. S. Avaliação da genotoxicidade de extratos de Boldo (Plectranthus ornatus) e Graviola (Annona muricata) através do Ensaio Cometa e do Teste de Micronúcleo em linfócitos humanos. Ph.D. Thesis, Brazilian Institute of Information in Science and Technology, 2016.

[ref63] Kwon M.; Leibowitz M. L.; Lee J.-H. Small but Mighty: The Causes and Consequences of Micronucleus Rupture. Exp. Mol. Med. 2020, 52 (11), 1777–1786. 10.1038/s12276-020-00529-z. 33230251 PMC8080619

[ref64] Ajsuvakova O. P.; Tinkov A. A.; Aschner M.; Rocha J. B. T.; Michalke B.; Skalnaya M. G.; Skalny A. V.; Butnariu M.; Dadar M.; Sarac I.; Aaseth J.; Bjørklund G. Sulfhydryl Groups as Targets of Mercury Toxicity. Coord. Chem. Rev. 2020, 417, 213343 10.1016/j.ccr.2020.213343. 32905350 PMC7470069

[ref65] Essex D. W. Redox Control of Platelet Function. Antioxid. Redox Signaling 2009, 11 (5), 1191–1225. 10.1089/ars.2008.2322. 19061441

[ref66] Kleffner I.; Eichler S.; Ruck T.; Schüngel L.; Pfeuffer S.; Polzer P.; Dittrich R.; Dziewas R.; Gross C. C.; Göbel K.; Wiendl H.; Kehrel B. E.; Meuth S. G. An Enigmatic Case of Acute Mercury Poisoning: Clinical, Immunological Findings and Platelet Function. Front. Neurol. 2017, 8, 517 10.3389/fneur.2017.00517. 29033890 PMC5625000

[ref67] Ribeiro R. F. L.; Germano A. Development and Validation of a Method for the Determination of Hg in Animal Tissues (Equine Muscle, Bovine Kidney and Swine Kidney, and Poultry Muscle) by Direct Mercury Analysis (DMA). Microchem. J. 2015, 121, 237–243. 10.1016/j.microc.2015.03.005.

[ref68] Rodrigues A. P. C.; Maciel P. O.; Silva L. C. C. P. da.; Albuquerque C.; Inácio A. F.; Freire M.; Linde A. R.; Almosny N. R. P.; Andreata J. V.; Bidone E. D.; Castilhos Z. C. Biomarkers for Mercury Exposure in Tropical Estuarine Fish. Ecotoxicology and Environmental Contamination 2010, 5( (1), ), 9−18.

[ref69] Silva J. da. O uso do ensaio cometa para o ensino de genética toxicológica. Genética na Escola 2007, 2 (2), 30–33. 10.55838/1980-3540.ge.2007.45.

[ref70] Gichner T.; Mukherjee A.; Velemínský J. DNA Staining with the Fluorochromes EtBr, DAPI and YOYO-1 in the Comet Assay with Tobacco Plants after Treatment with Ethyl Methanesulphonate, Hyperthermia and DNase-I. Mutat. Res., Genet. Toxicol. Environ. Mutagen. 2006, 605 (1–2), 17–21. 10.1016/j.mrgentox.2006.01.005. 16574466

[ref71] Gyori B. M.; Venkatachalam G.; Thiagarajan P. S.; Hsu D.; Clement M.-V. OpenComet: An Automated Tool for Comet Assay Image Analysis. Redox Biol. 2014, 2, 457–465. 10.1016/j.redox.2013.12.020. 24624335 PMC3949099

