# Supplementary material for: Genotoxic and Hematological Effects Associated with Chronic Dietary Mercury Toxicity in Juvenile Tilapia Oreochromis sp
Source: ACS Omega. 2025 Feb 20;10(8):8190–201. doi: 10.1021/acsomega.4c09749 (PMC11886740; doi:10.1021/acsomega.4c09749)
Supplement: Supplementary file 1 — ao4c09749_si_001.pdf [file ao4c09749_si_001.pdf]

Supporting Information

Genotoxic and hematological effects associated with chronic dietary mercury toxicity in juvenile tilapia *Oreochromis* sp.

Ahieska A. Liscano-Carreño[a,b,c], Cassiano J. Saatkamp[b,d], Ricardo B. de Oliveira[a,e], Luís R. R. Rodrigues[a,b,e].

[a] Programa de Pós-Graduação em Biodiversidade e Biotecnologia (REDE BIONORTE), Instituto de Saúde Coletiva (ISCO), Universidade Federal do Oeste do Pará (UFOPA), Rua Vera Paz, s/nº, Salé, CEP 68040-255, Santarém, Pará, Brazil.

[b] Laboratório de Genética & Biodiversidade (LGBio), Instituto de Ciências da Educação (ICED), Universidade Federal do Oeste do Pará (UFOPA), Rua Vera Paz, s/nº, Salé, CEP 68040-255, Santarém, Pará, Brazil.

[c] Departamento de Biología, Universidad de Oriente (UDO), Avenida Universidad, s/n°, Cod Postal 6101, Cumaná, Sucre, Venezuela

[d] Laboratório Santos, Santarém, PA, Brasil

[e] Instituto de Ciências da Educação (ICED), Universidade Federal do Oeste do Pará (UFOPA), Rua Vera Paz, s/nº, Salé, CEP 68040-255, Santarém, Pará, Brazil.

*Corresponding author: aye27leon@gmail.com

Summary table of biomarkers in juveniles Tilapia exposed to MeHg 0.5 $mg.kg^{-1}$. Relationship between variables associated with THg in juveniles exposed to MeHg 0.5 $mg.kg^{-1}$. Relative significance of variances of the variables associated with mercury bioaccumulation in blood of juvenile Tilapia up to 28 days of exposure. Juvenile tilapia in a bioassay of exposure to methylmercury by food intake. Certificate from the Animal Research Ethics Committee of the Federal University of Western Pará (UFOPA). Protocol for collecting organisms for research purposes SISBIO n° 86173-1.

Table S1. Summary table of biomarkers in juveniles Tilapia exposed to MeHg 0.5 mg.kg^{-1}. Presenting details of medians/median, standard desviation, KW/ANOVA and p-value.

	Grupo	t0 $\tilde{X}$	t0 SD	t7 $\tilde{X}$	t7 SD	t14 $\tilde{X}$	t14 SD	t21 $\tilde{X}$	t21 SD	t28 $\tilde{X}$	t28 SD	*KW*	*P*
THg m	CG	-	-	-	-	-	-	-	-	0.004	0.0009	11.36	0.0007
	EG	-	-	-	-	-	-	-	-	0.03	0.02		
Trb	CG	104.0	32.96	148.75	97.13	54.5	10.78	58.56	15.44	59.86	11.61	49.67	$1.24*10^{-7}$
	EG	101.0	23.33	140.0	34.74	75.86	29.59	61.56	11.36	51.5	18.42		
MCV	CG	252.52	154.14	396.14	148.13	201.76	27.90	231.88	65.57	258.06	123.16	15.28	0.08
	EG	243.9	86.56	226.42	29.04	193.65	72.96	224.14	36.47	294.12	129.57		

	Grupo	t0 X	t0 SD	t7 X	t7 SD	t14 X	t14 SD	t21 X	t21 SD	t28 X	t28 SD	Anova	*P*
THg s	CG	0.004	0.002	0.003	0.0002	0.004	0.003	0.0018	0.0001	0.001	0.0002	14.47	0.0001
	EG	0.014	0.009	0.014	0.0007	0.019	0.001	0.024	0.004	0.03	0.0054		
RBC	CG	0.96	036	0.82	0.51	1.29	0.20	1.05	0.28	0.94	0.27	1.99	0.05
	EG	1.14	0.38	1.12	0.26	1.28	0.29	1.26	0.19	1.03	0.31		
Hct%	CG	30.56	10.31	29.0	5.93	26.75	3.54	24.0	4.69	28.5	8.19	1.08	0.39
	EG	28.11	7.29	23.14	6.31	25.2	4.83	26.67	4.58	29.56	8.41		
Hgb	CG	5.74	2.78	6.15	1.55	7.98	1.86	8.49	2.27	7.89	0.98	3.45	0.001
	EG	5.63	1.43	6.8	1.41	8.1	1.56	8.89	1.24	6.24	2.57		
MCH	CG	49.59	21.66	67.32	13.57	61.94	8.69	82.26	16.33	89.11	22.84	4.68	0.0001
	EG	55.13	20.85	61.25	9.61	65.46	13.59	76.26	11.12	59.75	18.07		
MCHC	CG	18.62	8.48	21.15	8.52	30.20	7.05	35.13	4.93	31.01	10.38	5.44	0.000
	EG	21.16	6.57	29.02	3.41	32.39	2.97	33.63	3.60	23.39	12.34		

Table S2. Relationship between variables associated with THg in juveniles exposed to MeHg 0.5 $mg.kg^{-1}$. Spearman correlation

	Hct	Hgb	Trb	RBC	MN	Bn	Bug	Bl	Not	Erb	WBC
**Hct**	1	Ns	ns	0.4779<br>0.0025	ns	0.5825<br>0.0198	Ns	ns	ns	ns	ns
**Hgb**	ns	1	ns	0.4903<br>0.0017	ns	ns	Ns	ns	ns	0.5939<br>0.0263	0.5295<br>0.0476
**Trb**	ns	Ns	1	ns	-0.7202<br>0.0040	ns	-0.7448<br>0.0029	-0.8678<br>0.0005	-0.6785<br>0.0066	ns	ns
**RBC**	0.4779<br>0.0025	0.4903<br>0.0017	ns	1	ns	ns	Ns	ns	ns	0.7667<br>0.0030	0.7211<br>0.0052
**MN**	ns	Ns	-0.7202<br>0.0040	ns	1	0.5413<br>0.0256	0.8068<br>0.0009	0.8296<br>0.0006	0.8604<br>0.0004	ns	ns
**Bn**	0.5825<br>0.0198	Ns	ns	ns	0.5413<br>0.0256	1	Ns	0.6199<br>0.0106	0.6547<br>0.0069	ns	ns
**Bug**	ns	Ns	-0.7448<br>0.0029	ns	0.8068<br>0.0009	ns	1	0.7960<br>0.0010	0.7422<br>0.0022	ns	ns
**Bl**	ns	Ns	-0.8678<br>0.0005	ns	0.8296<br>0.0006	0.6199<br>0.0106	0.7960<br>0.0010	1	0.7691<br>0.0015	ns	ns
**Not**	ns	Ns	-0.6785<br>0.0066	ns	0.8604<br>0.0004	0.6547<br>0.0069	0.7422<br>0.0022	0.7691<br>0.0015	1	ns	ns
**Erb**	ns	0.5939<br>0.0263	ns	0.7667<br>0.0030	ns	ns	Ns	ns	ns	1	0.6882<br>0.0077
**WBC**	ns	0.5295<br>0.0476	ns	0.7211<br>0.0052	ns	ns	Ns	ns	ns	0.6882<br>0.0077	1

Table S3. Relative significance of variances of the variables associated with mercury bioaccumulation in blood of juvenile Tilapia up to 28 days of exposure.

	Component 1	*Component 2*
THg	0.37	0.33
Trb	-0.48	0.09
RBC	-0.23	0.51
Erb	-0.003	0.56
WBC	-0.27	0.50
MN	0.49	0.18
ENA	0.53	0.15

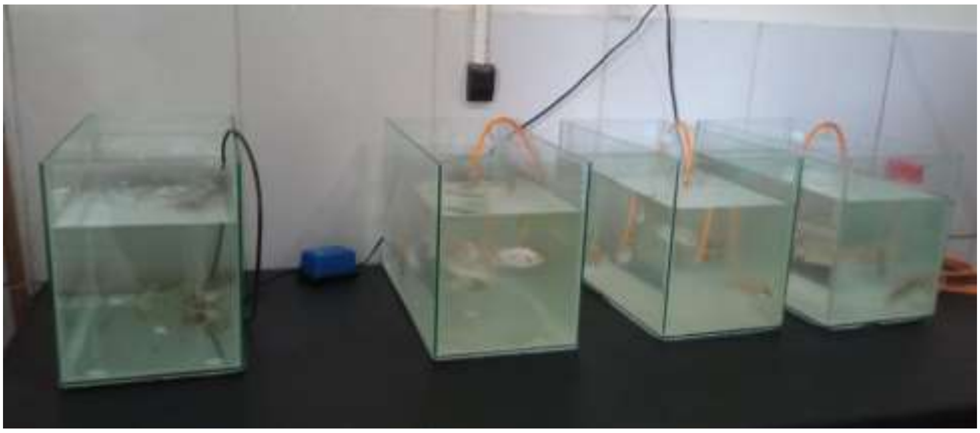

Figure S1: Juvenile tilapia in a bioassay of exposure to methylmercury by food intake. Distribution of fish in the aquariums and divided into the control group (CG) and the exposed group (EG).

Figure S2. Certificate from the Animal Research Ethics Committee of the Federal University of Western Pará

Universidade Federal do Oeste do Pará

COMISSÃO DE ÉTICA NO USO DE ANIMAIS

CEUA

CERTIFICADO

Certificamos que o Protocolo Nº **0520230254**, intitulado **AVALIAÇÃO DE BIOMARCADORES DE GENOTOXICIDADE EM PEIXES CONTAMINADOS POR MERCÚRIO NA BACIA DO RIO TAPAJÓS**, sob a responsabilidade de **Luís Reginaldo Ribeiro Rodrigues**, está de acordo com os Princípios éticos na Experimentação Animal adotados pelo Conselho Nacional de Controle de Experimentação Animal (CONCEA), tendo sido aprovado pela Comissão de Ética no Uso de Animais da Universidade Federal do Oeste do Pará - UFOPA.

CERTIFICATE

We certify that the protocol Nº **0520230254**, entitled **"AVALIAÇÃO DE BIOMARCADORES DE GENOTOXICIDADE EM PEIXES CONTAMINADOS POR MERCÚRIO NA BACIA DO RIO TAPAJÓS"**, is in agreement with the Ethical Principles for Animal Research established by the National Council for Control of Animal Experimentation (CONCEA). This project was approved by the institutional Commission for Ethics in the Use of Animals of Universidade Federal do Oeste do Pará.

Santarém-PA, 26/07/2023

Profª. Dra. Aline Pacheco
Presidente

Verificar a autenticidade do certificado em http://ufopa.edu.br/validar-certificado

Figure S3. Protocol for collecting organisms for research purposes SISBIO n° 86173-1

Ministério do Meio Ambiente - MMA

Instituto Chico Mendes de Conservação da Biodiversidade - ICMBio

Sistema de Autorização e Informação em Biodiversidade - SISBIO

Autorização para atividades com finalidade científica

Número: 86173-1	Data da Emissão: 11/01/2023 12:31:55	Data da Revalidação*: 11/01/2024

De acordo com o art. 28 da IN 03/2014, esta autorização tem prazo de validade equivalente ao previsto no cronograma de atividades do projeto, mas deverá ser revalidada anualmente mediante a apresentação do relatório de atividades a ser enviado por meio do Sisbio no prazo de até 30 dias a contar da data do aniversário de sua emissão.

Dados do titular

SISBIO

Nome: AHIESKA AIMARA LISCANO CARRENO	CPF: 717.374.551-96
Título do Projeto: EFEITOS DELETÉRIOS DA CONTAMINAÇÃO POR MERCÚRIO (HG) EM PEIXES NAS REGIÕES DE GARIMPO DE OURO NA BACIA DO RIO TAPAJÓS	
Nome da Instituição: UNIVERSIDADE FEDERAL DO OESTE DO PARÁ	CNPJ: 01.469.655/0001-76

Cronograma de atividades

#	Descrição da atividade	Início (mês/ano)	Fim (mês/ano)
1	Coletas de amostras e especimes de peixes, água e sedimentos	12/2022	12/2024

Equipe

#	Nome	Função	CPF	Nacionalidade
1	Luan Aércio Melo Maciel	Aluno de doutorado	020.260.022-07	Brasileira
2	Karen Larissa Auzier Guimarães	Aluna de doutorado	046.953.161-45	Brasileira
3	LUIS REGINALDO RIBEIRO RODRIGUES	pesquisador	391.501.272-68	Brasileira

Este documento foi expedido com base na Instrução Normativa nº Portaria ICMBio nº 748/2022. Através do código de autenticação abaixo, qualquer cidadão poderá verificar a autenticidade ou regularidade deste documento, por meio da página do Sisbio/ICMBio na Internet (www.icmbio.gov.br/sisbio).

Código de autenticação: 0861730120230111 **Página 1/5**

Ministério do Meio Ambiente - MMA

Instituto Chico Mendes de Conservação da Biodiversidade - ICMBio

Sistema de Autorização e Informação em Biodiversidade - SISBIO

Autorização para atividades com finalidade científica

Número: 86173-1	Data da Emissão: 11/01/2023 12:31:55	Data da Revalidação*: 11/01/2024
De acordo com o art. 28 da IN 03/2014, esta autorização tem prazo de validade equivalente ao previsto no cronograma de atividades do projeto, mas deverá ser revalidada anualmente mediante a apresentação do relatório de atividades a ser enviado por meio do Sisbio no prazo de até 30 dias a contar da data do aniversário de sua emissão.		

Dados do titular

Nome: AHIESKA AIMARA LISCANO CARRENO	CPF: 717.374.551-96
Título do Projeto: EFEITOS DELETÉRIOS DA CONTAMINAÇÃO POR MERCÚRIO (HG) EM PEIXES NAS REGIÕES DE GARIMPO DE OURO NA BACIA DO RIO TAPAJÓS	
Nome da Instituição: UNIVERSIDADE FEDERAL DO OESTE DO PARÁ	CNPJ: 01.469.655/0001-76

SISBIO

Observações e ressalvas

1	A autorização não eximirá o pesquisador da necessidade de obter outras anuências, como: I) do proprietário, arrendatário, posseiro ou morador quando as atividades forem realizadas em área de domínio privado ou dentro dos limites de unidade de conservação federal cujo processo de regularização fundiária encontra-se em curso; II) da comunidade indígena envolvida, ouvido o órgão indigenista oficial, quando as atividades de pesquisa forem executadas em terra indígena; III) do Conselho de Defesa Nacional, quando as atividades de pesquisa forem executadas em área indispensável à segurança nacional; IV) da autoridade marítima, quando as atividades de pesquisa forem executadas em águas jurisdicionais brasileiras; V) do Departamento Nacional da Produção Mineral, quando a pesquisa visar a exploração de depósitos fossilíferos ou a extração de espécimes fósseis; VI) do órgão gestor da unidade de conservação estadual, distrital ou municipal, dentre outras.
2	Deve-se observar as as recomendações de prevenção contra a COVID-19 das autoridades sanitárias locais e das Unidades de Conservação a serem acessadas.
3	Esta autorização NÃO libera o uso da substância com potencial agrotóxico e/ou inseticida e NÃO exime o pesquisador titular e os membros de sua equipe da necessidade de atender às exigências e obter as autorizações previstas em outros instrumentos legais relativos ao registro de agrotóxicos (Lei nº 7.802, de 11 de julho de 1989, Decreto nº 4.074, de 4 de janeiro de 2002, entre outros).
4	Esta autorização NÃO libera o uso da substância com potencial agrotóxico e/ou inseticida e NÃO exime o pesquisador titular e os membros de sua equipe da necessidade de atender às exigências e obter as autorizações previstas em outros instrumentos legais relativos ao registro de agrotóxicos (Lei nº 7.802, de 11 de julho de 1989, Decreto nº 4.074, de 4 de janeiro de 2002, entre outros).
5	As atividades de campo exercidas por pessoa natural ou jurídica estrangeira, em todo o território nacional, que impliquem o deslocamento de recursos humanos e materiais, tendo por objeto coletar dados, materiais, espécimes biológicos e minerais, peças integrantes da cultura nativa e cultura popular, presente e passada, obtidos por meio de recursos e técnicas que se destinem ao estudo, à difusão ou à pesquisa, estão sujeitas a autorização do Ministério de Ciência e Tecnologia.
6	Este documento somente poderá ser utilizado para os fins previstos na Portaria ICMBio nº 748/2022, no que especifica esta Autorização, não podendo ser utilizado para fins comerciais, industriais ou esportivos. O material biológico coletado deverá ser utilizado para atividades científicas ou didáticas no âmbito do ensino superior.
7	Este documento não dispensa o cumprimento da legislação que dispõe sobre acesso a componente do patrimônio genético existente no território nacional, na plataforma continental e na zona econômica exclusiva, ou ao conhecimento tradicional associado ao patrimônio genético, para fins de pesquisa científica, bioprospecção e desenvolvimento tecnológico. Veja maiores informações em www.mma.gov.br/cgen.
8	O titular de licença ou autorização e os membros da sua equipe deverão optar por métodos de coleta e instrumentos de captura direcionados, sempre que possível, ao grupo taxonômico de interesse, evitando a morte ou dano significativo a outros grupos; e empregar esforço de coleta ou captura que não comprometa a viabilidade de populações do grupo taxonômico de interesse em condição in situ.
9	Esta autorização NÃO exime o pesquisador titular e os membros de sua equipe da necessidade de obter as anuências previstas em outros instrumentos legais, bem como do consentimento do responsável pela área, pública ou privada, onde será realizada a atividade, inclusive do órgão gestor de terra indígena (FUNAI), da unidade de conservação estadual, distrital ou municipal, ou do proprietário, arrendatário, posseiro ou morador de área dentro dos limites de unidade de conservação federal cujo processo de regularização fundiária encontra-se em curso.
10	Em caso de pesquisa em UNIDADE DE CONSERVAÇÃO, o pesquisador titular desta autorização deverá contactar a administração da unidade a fim de CONFIRMAR AS DATAS das expedições, as condições para realização das coletas e de uso da infraestrutura da unidade.
11	O titular de autorização ou de licença permanente, assim como os membros de sua equipe, quando da violação da legislação vigente, ou quando da inadequação, omissão ou falsa descrição de informações relevantes que subsidiaram a expedição do ato, poderá, mediante decisão motivada, ter a autorização ou licença suspensa ou revogada pelo ICMBio, nos termos da legislação brasileira em vigor.

Este documento foi expedido com base na Instrução Normativa nº Portaria ICMBio nº 748/2022. Através do código de autenticação abaixo, qualquer cidadão poderá verificar a autenticidade ou regularidade deste documento, por meio da página do Sisbio/ICMBio na Internet (www.icmbio.gov.br/sisbio).

Ministério do Meio Ambiente - MMA
Instituto Chico Mendes de Conservação da Biodiversidade - ICMBio
Sistema de Autorização e Informação em Biodiversidade - SISBIO

Autorização para atividades com finalidade científica

Número: 86173-1	Data da Emissão: 11/01/2023 12:31:55	Data da Revalidação*: 11/01/2024

De acordo com o art. 28 da IN 03/2014, esta autorização tem prazo de validade equivalente ao previsto no cronograma de atividades do projeto, mas deverá ser revalidada anualmente mediante a apresentação do relatório de atividades a ser enviado por meio do Sisbio no prazo de até 30 dias a contar da data do aniversário de sua emissão.

Dados do titular

SISBIO

Nome: AHIESKA AIMARA LISCANO CARRENO	CPF: 717.374.551-96
Título do Projeto: EFEITOS DELETÉRIOS DA CONTAMINAÇÃO POR MERCÚRIO (HG) EM PEIXES NAS REGIÕES DE GARIMPO DE OURO NA BACIA DO RIO TAPAJÓS	
Nome da Instituição: UNIVERSIDADE FEDERAL DO OESTE DO PARÁ	CNPJ: 01.469.655/0001-76

Outras ressalvas

1		CEPAM Manaus-AM

Locais onde as atividades de campo serão executadas

#	Descrição do local	Município-UF	Bioma	Caverna?	Tipo
1	Garimpo Creporizão - Rio Crepori	Itaituba-PA	Amazônia	Não	Fora de UC Federal
2	Vila Creporizinho - Rio Creporizinho	Itaituba-PA	Amazônia	Não	Fora de UC Federal
3	Garimpo São Chico	Itaituba-PA	Amazônia	Não	Fora de UC Federal
4	várzeas do baixo Amazonas	Santarém-PA	Amazônia	Não	Fora de UC Federal
5	Rio Tapajós e tributários	Jacareacanga-PA	Amazônia	Não	Fora de UC Federal

Atividades

#	Atividade	Grupo de Atividade
1	Coleta/transporte de amostras biológicas in situ	Fora de UC Federal
2	Captura de animais silvestres in situ	Fora de UC Federal
3	Manutenção temporária (até 24 meses) de vertebrados silvestres em cativeiro	Atividades ex-situ (fora da natureza)
4	Coleta/transporte de espécimes da fauna silvestre in situ	Fora de UC Federal

Atividades X Táxons

#	Atividade	Táxon	Qtde.
1	Coleta/transporte de espécimes da fauna silvestre in situ	Hoplias	20
2	Captura de animais silvestres in situ	Hoplias	-
3	Coleta/transporte de amostras biológicas in situ	Hoplias	-
4	Coleta/transporte de espécimes da fauna silvestre in situ	Serrasalmus	20
5	Captura de animais silvestres in situ	Serrasalmus	-
6	Coleta/transporte de amostras biológicas in situ	Serrasalmus	-
7	Coleta/transporte de espécimes da fauna silvestre in situ	Oreochromis	20
8	Manutenção temporária (até 24 meses) de vertebrados silvestres em cativeiro	Oreochromis	-
9	Captura de animais silvestres in situ	Oreochromis	-
10	Coleta/transporte de amostras biológicas in situ	Oreochromis	-

A quantidade prevista só é obrigatória para atividades do tipo "Coleta/transporte de espécimes da fauna silvestre in situ". Essa quantidade abrange uma porção territorial mínima, que pode ser uma Unidade de Conservação Federal ou um Município.

Este documento foi expedido com base na Instrução Normativa nº Portaria ICMBio nº 748/2022. Através do código de autenticação abaixo, qualquer cidadão poderá verificar a autenticidade ou regularidade deste documento, por meio da página do Sisbio/ICMBio na Internet (www.icmbio.gov.br/sisbio).

Ministério do Meio Ambiente - MMA
Instituto Chico Mendes de Conservação da Biodiversidade - ICMBio
Sistema de Autorização e Informação em Biodiversidade - SISBIO

Autorização para atividades com finalidade científica

Número: 86173-1	Data da Emissão: 11/01/2023 12:31:55	Data da Revalidação*: 11/01/2024
De acordo com o art. 28 da IN 03/2014, esta autorização tem prazo de validade equivalente ao previsto no cronograma de atividades do projeto, mas deverá ser revalidada anualmente mediante a apresentação do relatório de atividades a ser enviado por meio do Sisbio no prazo de até 30 dias a contar da data do aniversário de sua emissão.		

Dados do titular

Nome: AHIESKA AIMARA LISCANO CARRENO	CPF: 717.374.551-96
Título do Projeto: EFEITOS DELETÉRIOS DA CONTAMINAÇÃO POR MERCÚRIO (HG) EM PEIXES NAS REGIÕES DE GARIMPO DE OURO NA BACIA DO RIO TAPAJÓS	
Nome da Instituição: UNIVERSIDADE FEDERAL DO OESTE DO PARÁ	CNPJ: 01.469.655/0001-76

A quantidade significa: por espécie X localidade X ano.

Materiais e Métodos

#	Tipo de Método (Grupo taxonômico)	Materiais
1	Amostras biológicas (Peixes)	Fragmento de tecido/órgão, Sangue
2	Método de captura/coleta (Peixes)	Anzol e linha (op.manual):linha de mão,de corso,carretilha,molinete,corrico,vara e isca viva, Rede de emalhar (emalhe de deriva, de fundo, malhadeiras, caceio, feiticeiras,tresmalhos e caçoeira), Tarrafa

Destino do material biológico coletado

#	Nome local destino	Tipo destino
1	UNIVERSIDADE FEDERAL DO OESTE DO PARÁ	Laboratório

Este documento foi expedido com base na Instrução Normativa nº Portaria ICMBio nº 748/2022. Através do código de autenticação abaixo, qualquer cidadão poderá verificar a autenticidade ou regularidade deste documento, por meio da página do Sisbio/ICMBio na Internet (www.icmbio.gov.br/sisbio).

Código de autenticação: 0861730120230111

Ministério do Meio Ambiente - MMA

Instituto Chico Mendes de Conservação da Biodiversidade - ICMBio

Sistema de Autorização e Informação em Biodiversidade - SISBIO

Autorização para atividades com finalidade científica

Número: 86173-1	Data da Emissão: 11/01/2023 12:31:55	Data da Revalidação*: 11/01/2024

De acordo com o art. 28 da IN 03/2014, esta autorização tem prazo de validade equivalente ao previsto no cronograma de atividades do projeto, mas deverá ser revalidada anualmente mediante a apresentação do relatório de atividades a ser enviado por meio do Sisbio no prazo de até 30 dias a contar da data do aniversário de sua emissão.

Dados do titular

SISBIO

Nome: AHIESKA AIMARA LISCANO CARRENO	CPF: 717.374.551-96
Título do Projeto: EFEITOS DELETÉRIOS DA CONTAMINAÇÃO POR MERCÚRIO (HG) EM PEIXES NAS REGIÕES DE GARIMPO DE OURO NA BACIA DO RIO TAPAJÓS	
Nome da Instituição: UNIVERSIDADE FEDERAL DO OESTE DO PARÁ	CNPJ: 01.469.655/0001-76

Registro de coleta imprevista de material biológico

De acordo com a Instrução Normativa nº03/2014, a coleta imprevista de material biológico ou de substrato não contemplado na autorização ou na licença permanente deverá ser anotada na mesma, em campo específico, por ocasião da coleta, devendo esta coleta imprevista ser comunicada por meio do relatório de atividades. O transporte do material biológico ou do substrato deverá ser acompanhado da autorização ou da licença permanente com a devida anotação. O material biológico coletado de forma imprevista, deverá ser destinado à instituição científica e, depositado, preferencialmente, em coleção biológica científica registrada no Cadastro Nacional de Coleções Biológicas (CCBIO).

Táxon*	Qtde.	Tipo de Amostra	Qtde.	Data

* Identificar o espécime do nível taxonômico possível.

Este documento foi expedido com base na Instrução Normativa nº Portaria ICMBio nº 748/2022. Através do código de autenticação abaixo, qualquer cidadão poderá verificar a autenticidade ou regularidade deste documento, por meio da página do Sisbio/ICMBio na Internet (www.icmbio.gov.br/sisbio).

Código de autenticação: 0861730120230111
